# Impact of process parameters on IgG glycosylation in CHO systems: a comprehensive quantitative analysis

**DOI:** 10.1080/19420862.2026.2643039

**Published:** 2026-03-15

**Authors:** Javier Bravo-Venegas, Jose Rodriguez-Siza, Mauricio Vergara, Mauro Torres, Alan Dickson, Jorge R. Toledo, María Carmen Molina, Marcela A. Hermoso, Julio Berríos, Claudia Altamirano

**Affiliations:** a Department of Chemical Engineering, University of Manchester, Manchester, UK; b Manchester Institute of Biotechnology, University of Manchester, Manchester, UK; c School of Biochemical Engineering, Faculty of Engineering, Pontificia Universidad Católica de Valparaíso, Valparaíso, Chile; d Center for Interdisciplinary Research in Biomedicine, Biotechnology and Well-Being (CID3B), Pontificia Universidad Católica de Valparaíso, Valparaíso, Chile; e School of Chemical Engineering, The University of Queensland, Brisbane, Australia; f Laboratorio de biotecnología y Biofarmacia, Departamento de Fisiopatología, Facultad de Ciencias Biológicas, Universidad de Concepción, Concepción, Chile; g Laboratorio de anticuerpos recombinates e inmuno-oncologia, Núcleo Interdisciplinario de Farmacología e Inmunología (NIFI), Instituto de Ciencias Biomédicas (ICBM), Facultad de Medicina, Universidad de Chile, Santiago, Chile; h Immunology Programme, Faculty of Medicine, University of Chile, Santiago, Chile; i Department of Gastroenterology and Hepatology, University of Groningen, Groningen, The Netherlands; j Centro Regional de Estudio en Alimentos Saludables, Valparaíso, Chile; k IMPACT, Center of Interventional Medicine for Precision and Advanced Cellular Therapy, Santiago, Chile

**Keywords:** Monoclonal antibodies, CHO cells, critical quality attributes (CQA), glycan indices, glycan distribution, culture conditions, bioprocess optimization, quantitative analysis, systematic review, therapeutic efficacy

## Abstract

Controlling glycosylation, a critical quality attribute of biopharmaceuticals such as monoclonal antibodies, is essential, as it significantly influences biological activity and therapeutic efficacy. Although numerous studies have examined the impact of process parameters (PP, e.g. temperature, pH, dissolved oxygen) on glycosylation, the lack of standardized reporting makes cross-study comparisons challenging and prevents clear conclusions. Here, we systematically reviewed the literature and applied a normalized quantitative framework, the Glycan Indices approach, as a standardized quantitative criterion to evaluate the impact of process parameters on glycoform distribution in IgG-producing CHO cell systems objectively. This methodology enabled the integration and reinterpretation of large, heterogeneous datasets, validating some well-known patterns while providing novel perspectives about process parameters. Our analysis revealed that PP manipulations of pH, dissolved oxygen or CO_2_ partial pressure rarely resulted in meaningful shifts in glycosylation, with changes <5% observed for galactose, fucose, or N-acetylneuraminic acid content. In contrast, for several cases temperature and osmolality changes notably affected galactosylation (>10%) and fucosylation (1–10%), variations that may have significant biological consequences. To our knowledge, this is the first comprehensive quantitative assessment of process parameters effects on glycosylation, showing that such influences are consistently limited, independent of CHO cell line or culture mode. Based in our observations we strongly recommend reporting both glycan distribution and glycan indices when performing glycan analysis. Dual reporting facilitates inter-study comparisons and prevents subtle shifts in sugar moieties from being masked by glycan redistribution.

## Introduction

Monoclonal antibodies (mAbs) have become unique therapeutic tools of modern medicine, offering treatment of a wide variety of complex diseases, including cancers,^1^ autoimmune disorders and infectious diseases.^2^ Their therapeutic value lies in their high specificity and effectiveness in targeting specific antigens. By binding their targets, mAbs can exert various functions, such as blocking cytokine signals, modulating immunity and promoting cell death.^3^ The latest developments of modified mAb molecules, such as antibody-drug conjugates and multi-specifics, further expands their therapeutic potential and effectiveness.^4^ With their therapeutic efficacy, mAbs have achieved remarkable economic success, leading the list of best-selling drugs worldwide.^5^ Despite their successes, industrial mAb development is has challenges, with glycosylation as a significant area.

Due to their molecular complexity, mAbs are currently produced primarily in mammalian systems, with the Chinese hamster ovary (CHO) cell lineage providing the gold standard host.^6^ In part, CHO cell lines are preferred because they possess the cellular machinery able to perform human-like post-translational modifications (PTMs, a key structural aspect to ensure product biosafety with therapeutic delivery.

**Figure 1.**
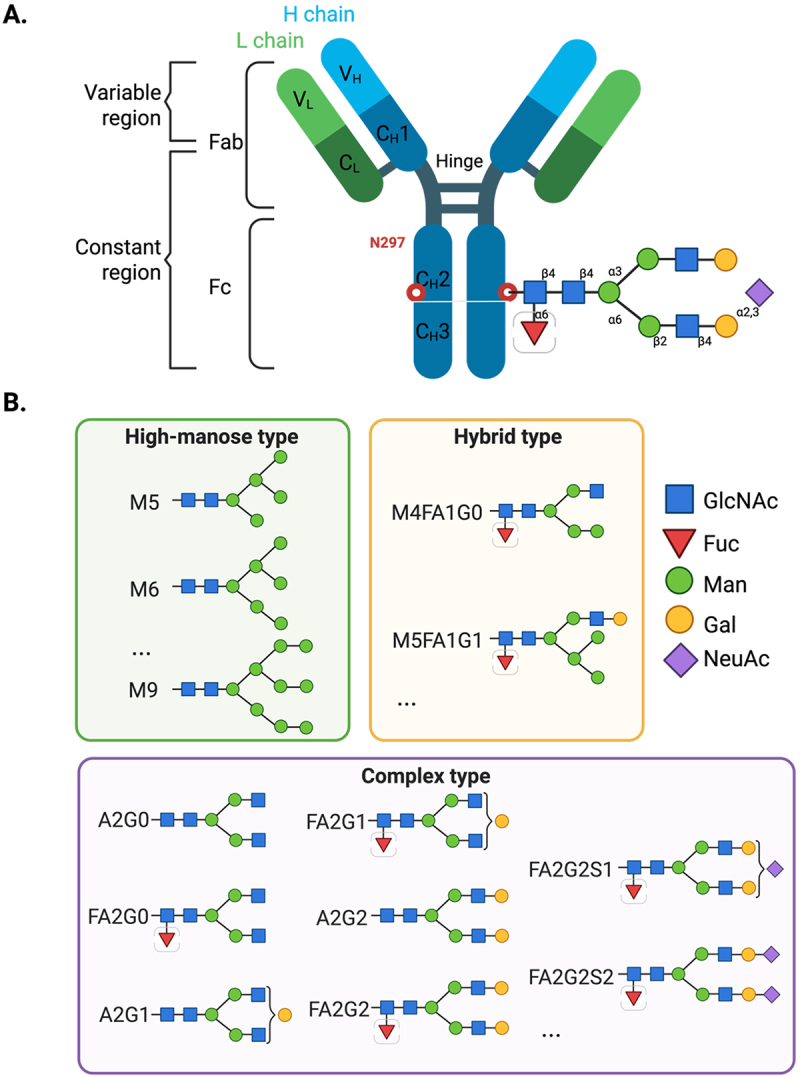
(A). General overview of an IgG antibody structure formed by four polypeptide chains, two heavy (H) (in blue) and two light (L) (in green), joined by S-S bonds. At the N-terminus of each chain, is found the V_H_ and V_L_ variable domains of the H and L chains, respectively. This combination forms the antigen binding site. The conserved N297 glycosylation site located in the C_H_2 of each H chain is depicted in red. Here, an example of an N-glycan structure for CHO is shown. (B). Common IgG glycan motifs, with particular emphasis on the complex-type N-glycans, that are important in the production of mAbs in CHO platforms are shown. For species high-mannose, there may be high-manose type variants from M5 to M9, including M6, M7 and M8. Adapted from Carrara et al.^8^ *Abbreviations*: A, antennarity; fab, antigen binding fragment; Fc, crystallizable fragment; GlcNAc, N-acetylglucosamine; fuc, fucose; F, fucose occupancy; man, manose; M, mannose occupancy; Gal, galactose; G, galactose occupancy; NeuAc, N-acetylneuraminic acid, S, NeuAc occupancy. Created with BioRender.com.

Glycosylation, a PTM, is a critical quality attribute (CQA) that can influence biological functions, stability, folding, immunogenicity and effector functions of therapeutic mAbs.^7^ The Fc domain of immunoglobulin G (IgG) antibodies, crucial for mediating effector functions, has a single conserved N-glycosylation site commonly located at residue 297 of the heavy chain (Figure 1A).^8^ The challenge is not the presence of glycan per se but the heterogeneity in glycan composition/structure that may arise due to variation in control conditions during manufacture, influencing their efficacy, stability and effector function profiles.^9,10^ Factors such as the choice of the host cell line, cell culture conditions, and nutrient supply can significantly impact the glycosylation patterns.^11^

Based on their structure, the glycan motifs have been classified into different groups, with recognizable properties (Figure 1B).^12^ For instance, high mannose glycans have been shown to increase the rate of serum clearance,^13^ while the presence or absence of fucose on the Fc glycan can strongly affect antibody-dependent cellular toxicity (ADCC).^14^ Additionally, galactosylation and sialylation levels can influence effector functions and anti-inflammatory activity.^15,16^ As these effects are product-specific, it is challenging to define a universally “optimal” glycosylation form, as the desired glycoform depends on the intended therapeutic function of the antibody.

To relate structural glycosylation differences to biological outcomes, Fc glycosylation must be considered as a key determinant of antibody effector functions. The Fc region mediates complement-dependent cytotoxicity (CDC) and FcγR-dependent activities, including ADCC, phagocytosis (ADCP) and trogocytosis (ADCT).^1^ These functions depend on the Fc binding to complement factor (C1q) or specific FcγRs on immune cells, and are strongly influenced by Fc glycan pattern.^17^ Consideration must also be given to non-glycosylated IgG species and asymmetric Fc glycosylation, which can modulate FcγR binding and downstream effector responses.^18^ These findings highlight the relevance of Fc-level heterogeneity beyond average glycan composition.

Because Fc glycosylation directly influences biological activity, its control becomes particularly critical in the context of biopharmaceutical development and regulatory approval. A consistent and well-defined glycosylation profile is particularly relevant in the development of biosimilars, which must demonstrate a high degree of similarity to the original product, including their glycosylation profile, to avoid clinically meaningful differences.^19^ Glycoengineering strategies have advanced in both genetic modifications and additives supplementation. For instance, knocking in, out or down genes encoding specific glycosyltransferases and nucleotide sugar transporters has yielded CHO cells capable of producing mAbs favoring specific glycoforms.^20^ Alternatively, supplementation of cell culture media with compounds, such as nucleotide-sugar precursors (Uridine, N-acetylmannosamine), trace metals (Mn, Zn) or other sugars (galactose), has been shown to offer the potential for simpler routes to adjust product glycosylation without extensive cell engineering.^21,22^

A less explored route involves manipulating the manufacturing process. Temperature shifts, pH, dissolved oxygen (DO), carbon dioxide partial pressure (*p*CO_2_), osmolality or small-molecule additives (e.g. sodium butyrate (NaBu)) can alter glycosylation machinery and downstream glycan profiles. However, their effects are highly context-dependent, varying with cell line, product type, and culture conditions.^23^ Maintaining stable process conditions is complicated by parameter fluctuations during bioprocessing, making consistent glycosylation challenging.

**Figure 2.**
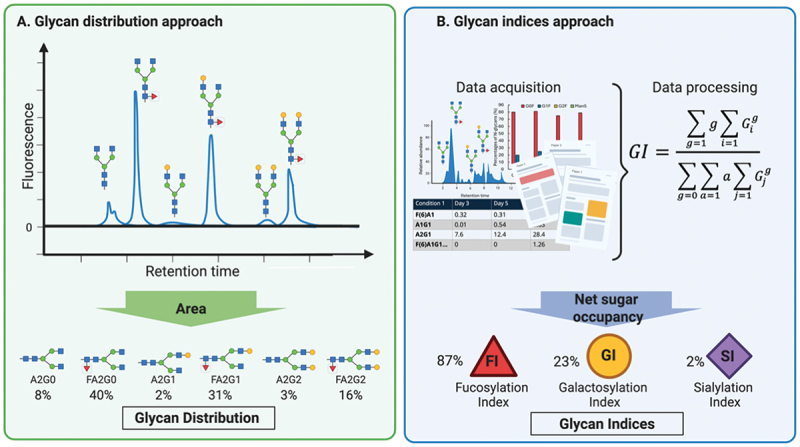
Common approaches to report the glycan analysis in literature, including the glycan distribution (A) obtained by HPLC/MS and the glycan indices calculation (B) according to the methodology proposed by Blondeel & Aucoin.^26^ *Abbreviations*: FI, fucosylation index; GI, galactosylation index; SI, sialylation index. Created with BioRender.com.

The scientific literature presents different approaches for reporting mAb glycosylation analysis.^24,25^ A standardized framework for data presentation would benefit the sector. The most frequently employed methods are the glycan distribution approach and the Glycan Index (GIx) approach. In the former, the relative abundance of individual glycoforms within the glycan pool is reported as a percentage (Figure 2A). In contrast, the GIx approach quantifies the net sugar occupancy for specific monosaccharides, typically fucose, galactose, and/or NeuAc, within the overall glycan distribution (Figure 2B). Calculating the net percentage of monosaccharide occupancy, this approach integrates glycan distribution data with weighting equations that estimate the percentage of potential glycosylation sites occupied by each specific sugar residue.^26–29,30–34^ The GIx calculation method proposed by Blondeel & Aucoin^26^ stands out for its clarity, robustness and reproducibility, defining a set of coefficients for each of the possible glycoforms, leaving no room to ambiguous calculations for each index.

This work aims to quantitatively evaluate the impact of key process parameters (PPs) on glycosylation. To date, no previous review study has used a standardized quantitative criterion to compare the impact of PP on glycosylation across works. Previous reviews have been purely descriptive,^35^ making it difficult to draw conclusions and impeding an objective comparison between experiments. To enable cross-study comparisons in culture parameters effect on glycosylation, we applied the GIx framework for a unified metric methodology for fucosylation (FI), galactosylation (GI), and sialylation (SI) indices. Here, the GIx approach is extended beyond its original use in culture media supplementation to encompass PPs, thereby covering the full range of culture environmental factors and providing new perspectives on previously published results. PPs alone proved to be insufficient to control glycosylation, whereas standardized GIx approach enable clearer insights and better process design.

## Materials & methods

### Data sources and search strategy

The compilation of peer-reviewed literature was conducted using indexed journal databases (e.g., Scopus, Web of Science, PubMed, Wiley Online Library, and Google Scholar) for records published from 2010 to 2024.

### Eligibility criteria and study selection

Studies were considered eligible if they (1) used CHO cells as production system, (2) produced an IgG-type mAb or related product, (3) reported Fc N-glycan measurements as glycoform distributions or GIx, and (4) investigated at least one PP expected to influence glycosylation. In total, 39 articles were identified as meeting these criteria, of which 32 provided sufficient data for GIx quantitative analysis. Among these articles a total of 78 PP manipulations were identified. Rationales for exclusion included absence of glycan data, non-IgG products, non-CHO hosts or insufficient detail to compute indices. Non-CHO systems were excluded because of lower market relevance and potential differences in glycosylation patterns, which could mislead interpretation.

### Data extraction and quality assessment

Articles presenting glycoform distribution or GIx data relevant to a specific PPs were used for analysis. Experimental data reporting GIx or data that enabled its calculation were extracted from the selected articles.

### Glycan indices calculation

FI, GI, and SI glycan indices were calculated following Blondeel & Aucoin.^26^ The mannose index was excluded because most articles did not report the data need for its calculation. Equations reflect the percentage of occupied fucose, galactose, or sialic acid sites based on the glycan distribution. For each moiety, the GIx was calculated according to the following equations:

(1) $$\mathrm{GI}=\frac{\sum_{g=1}g\sum_{i=1}G_{i}^{g}}{\sum_{g=0}\sum_{a=1}a\sum_{j=1}G_{j}^{g}}\times 100$$

(2) $$\mathrm{SI}=\frac{\sum_{s=1}s\sum_{i=1}G_{i}^{s}}{\sum_{s=0}\sum_{g=1}g\sum_{j=1}G_{j}^{s}}\times 100$$

(3) $$\mathrm{FI}=\frac{\sum_{f=1}f\sum_{i=1}G_{i}^{f}}{\sum_{f=0}\sum_{j=1}G_{j}^{f}}\times 100$$

Where:

*G*_i_^*^ is the portion of glycoforms with a particular moiety (g, s and f) and

*G*_j_^*^ is the portion of glycoforms able to receive a particular moiety.

*g* is the number of terminal galactose moieties per glycan

*a* is the number of terminal GlcNAc moieties per glycan.

*s* is the number of terminal sialic acid moieties per glycan.

*f* is the binary number of core *α*_*(1*→*6*)_ fucosylation per glycan.

After calculating the GIx, data were clustered by operational parameter, then by perturbation type (e.g., increase/decrease or presence/absence), culture mode and CHO cell lineage. ΔGIx was defined as the absolute percentage difference between control and manipulated conditions. Summative indices for each sugar motif (i.e., fucosylation, galactosylation and sialylation), were calculated to evidence global changes in distributions and reported in the Supplementary appendix. Note that in the case of fucose, the results were presented as a summative index for fucose, to continue the trend presented for the fucose index (FI). Naturally, the sum of afucosylates would correspond to the difference to complete 100%. The summative indices were calculated according to the following equations, which take into account all pertinent species with galactose, fucose or sialic acid motifs respectively:

(4) $$\mathrm{Summ}\;\mathrm{index}\;\mathrm{galactose}=\underset{i=1}{\sum }G_{i}^{g}$$

(5) $$\mathrm{Summ}\;\mathrm{index}\;\mathrm{sialic}\;\mathrm{acid}=\underset{i=1}{\sum }G_{i}^{s}$$

(6) $$\mathrm{Summ}\;\mathrm{index}\;\mathrm{fucose}=\underset{i=1}{\sum }G_{i}^{f}$$

Where:

*G*_i_^*^ is the portion of glycoforms with a particular moiety (g, s and f).

ΔSummative index was defined as the absolute percentage difference between control and manipulated conditions. For studies that reported only GIx without providing full glycan distributions, the published GIx were used as reported by the authors. In these cases, summative indices could not be calculated. An example of the calculations is provided in the Supplementary methods.

## Results

### Overview of mAb glycosylation data

We reviewed publicly available studies that investigated the impact of PPs on a mAb’s glycosylation and grouped accordingly. For each study, we identified the glycan analysis approach and if the GIx were not reported, determined whether the reported data were sufficient to calculate them. We found that most manipulations (54 of 78) did not report glycan distribution and GIx together (Tables 1–5). Among the 26 conditions that reported GIx, 17 provided only GIx without the underlying glycan distribution. In contrast, 37 conditions reported only individual glycoform species, and 15 provided no detailed mAb glycan distribution data.

**Table 1.** Reported effect of culture temperature manipulations on glycan distribution, including glycoform distribution and glycan indices, of therapeutic IgG-related products in CHO cell systems.

Type of manipulation		Cell line	Product	Culture mode	Culture timeline	Reported effect		Suitable for calculating glycan indices	Reference
						On glycoform distribution	On net content		
***Temperature***									
**Constant higher setpoint**									
	38, 39°C	CHO DUXB	EPO-Fc	Batch	5 L bioreactor	Not reported	↓DoS	✗	Trummer et al.^36^
**Constant lower setpoint**									
	32°C	CHO-K1	IgG4	Batch	Shake flask	↑FA2G0, ↓FA2G1	Not reported	✓	Galbraith et al.^25^
	33°C	CHO DUXB	Eg2-hFC	Batch	250 mL shake flask	↓FA2G0, ↓A2G1, ↑FA2G1, ↓A2G2, ↑FA2G1	Not reported FI, no effect on GI, SI	✓	Aghamohseni et al.^24^
	30, 33, 35°C	CHO DUXB	EPO-Fc	Batch	5 L bioreactor	Not reported	⇅DoS	✗	Trummer et al.^36^
	33°C	CHO DG44	IgG	Batch	125 mL shake flask	↓FA2G0, ↑FA2G1S1	Not reported	✓	Kim et al.^23^
**Shift to lower setpoint**									
	37→30°C	CHO	mAb	Batch	100 mL spinner flask	Not reported	↓SAC	✗	Chen et al.^37^
	37→33°C	CHO DUXB	Eg2-hFC	Batch	250 mL shake flask, shift at day 3	↑FA2G0, ↓A2G1, ↓FA2G2	Not reported FI, no effect on SI, ↓GI	✓	Aghamohseni et al.^24^
	37→30, 33°C	CHO DG44	IgM	Batch	1.5 L bioreactor, shift at day 2	Not reported	No effect on FI and SI	Taken from the article	Hennicke et al.^29^
	37→33°C	CHO DG44	IgG	Batch	125 mL shake flask, shift at day 4	↑FA2G1S1, ↓FA2G2S2	Not reported	✓	Kim et al.^23^
	37→30, 34°C	CHO GS	IgG1	Batch	250 mL bioreactor, shift at day 3	Not reported	No effect on FI, GI	Taken from the article	Madabhushi et al.^38^
	37→32°C	CHO GS	IgG4	Batch	3.5 L bioreactor, shift at day 6	↑A2G0, ↓FA2G0, ↓A2G1, ↓FA2G1	Not reported	✓	Tait et al.^39^
	36.5→33.5, 35°C	CHO	IgG1	Fed-batch	50 mL culture, shift at day 5	↑FA2G0, ↓A2G1, ↓FA2G1, ↓FA2G2	Not reported	✓	Yang & Ierapetritou^40^
	37→32, 34°C	CHO-K1	IgG1	Fed-batch	2.2 L bioreactor	No effect on glycoforms	Not reported	✗	St. Amand et al.^41^
	36.5→32°C	CHO T	IgG	Fed-batch	1.5 L bioreactor, shift at day 6	↓FA2G1	Not reported	✓	Sou et al.^42^
	36.5→32°C	CHO T	IgG	Fed-batch	1.5 L bioreactor, shift at day 6	↓FA2G1	Not reported	✓	Sou et al.^43^
	37→32°C	CHO DG44	IgG-fusion	Fed-batch	5 L bioreactor	Not reported	No effect on SAC	✗	Jing et al.^44^
	34→30°C	CHO GS	IgG	Fed-batch	2 L bioreactor, shift at day 5	↑A2G1, ↓FA2G2, ↓FA2G2S1,	No effect on FI, GI, SI	✓	Mellahi et al.^31^
	37→32°C	CHO GS	IgG1	Fed-batch	5 L bioreactor, shift at day 5	↑A2G0, ↑FA2G0, ↓FA2G1	Not reported	✓	McHugh et al.^45^
**Periodic shift**									
	37↔33°C	CHO DG44	IgG	Batch	125 mL shake flask, shift every 24, 48 h	↓FA2G0	Not reported	✓	Kim et al.^23^

Abbreviations: DoS, degree of sialylation [mol sialic acid/mol EPO-Fc]; FI, fucosylation index; GI, galactosylation index; SAC, sialic acid content [%]; SI, sialylation index.

**Table 2.** Reported effect of culture pH manipulations on glycan distribution, including glycoform distribution and glycan indices, of therapeutic IgG-related products in CHO cell systems.

Type of manipulation		Cell line	Product	Culture mode	Culture timeline	Reported effect		Suitable for calculating glycan indices	Reference
						On glycoform distribution	On net content		
***pH***									
**Without pH control**									
	No control	CHO	IgG	Batch	Shake flask	Not reported	↑FI, no effect on GI	✓	Maralingannavar et al.^30^
	No control	CHO DG44	IgG	Batch	125 mL shake flask	↓FA2G0, ↑FA2G1, ↑FA2G2	Not reported	✓	Kim et al.^23^
	No control	CHO DG44	IgG1	Batch	3 L bioreactor	↑FA2G0, ↓FA2G1, ↓FA2G2	Not reported FI, SI, No effect on GI	✓	Monteil et al.^32^
	No control	CHO DG44	IgG1	Batch	5 L shake bottle	↑FA2G0, ↓FA2G1, ↓FA2G2	Not reported FI, SI, ↓GI	✓	Monteil et al.^32^
**Constant higher setpoint**									
	7.2	CHO	mAb	Batch	3 L bioreactor	Not reported	No effect on FI, ↑GI,↑SI	Taken from the article	Brunner et al.^28^
	7.1, 7.2, 7.3	CHO DUXB	EPO-Fc	Batch	5 L bioreactor	Not reported	↓DoS	✗	Trummer et al.^36^
	7.2, 7.4	CHO DG44	IgG	Batch	125 mL shake flask	↓FA2G0, ↑FA2G1, ↑FA2G2	Not reported	✓	Kim et al.^23^
	7.2	CHO GS	mAb	Batch	250 mL bioreactor	↓FA2G0, ↑FA2G1, ↑FA2G2	No effect on FI, GI	✓	Madabhushi et al.^38^
	7.3	CHO-K1	IgG1	Fed-batch	2.2 L bioreactor	⇅FA2G1, ⇅A1G0, ⇅M3	Not reported	✗	St. Amand et al.^41^
	7.1	CHO DG44	IgG-fusion	Fed-batch	5 L bioreactor	Not reported	No effect on SAC	✗	Jing et al.^44^
	7.1	CHO GS	IgG	Fed-batch	2 L bioreactor	↓FA2G0, ↑FA2G1, ↑FA2G2	Not reported	✓	Lee et al.^46^
	7.03–7.24	CHO GS	IgG1	Fed-batch	250 mL bioreactor	Not reported	Not reported FI, SI, ⇅(FA2G1+FA2G2)	Taken from the article	Jiang et al.^47^
**Constant lower setpoint**									
	6.8	CHO	mAb	Batch	3 L bioreactor	Not reported	No effect on FI, SI, ↓GI	Taken from the article	Brunner et al.^28^
	6.8	CHO DUXB	Eg2-hFC	Batch	500 mL shake flask	↓FA2G0, ↓FA2G1, ↑FA2G2	Not reported FI, ↑GI,↑SI	✓	Aghamohseni et al.^27^
	6.8, 6.9	CHO DUXB	EPO-Fc	Batch	5 L bioreactor	Not reported	↓DoS	✗	Trummer et al.^36^
	6.6, 6.8	CHO DG44	IgG	Batch	125 mL shake flask	↑FA2G0, ↓FA2G1, ↑FA2G2	Not reported	✓	Kim et al.^23^
	6.8	CHO GS	mAb	Batch	250 mL bioreactor	Not reported	No effect on FI, GI	Taken from the article	Madabhushi et al.^38^
	6.8	CHO-K1	IgG1	Fed-batch	2.2 L bioreactor	⇅FA2G1, ⇅A1G0, ⇅M3	Not reported	✗	St. Amand et al.^41^
	6.7	CHO GS	IgG	Fed-batch	2 L bioreactor	↓FA2G1, ↓FA2G2	Not reported	✓	Lee et al.^46^
	6.78–6.97	CHO GS	IgG1	Fed-batch	250 mL bioreactor	Not reported	Not reported FI, SI, ↓(FA2G1+FA2G2)	Taken from the article	Jiang et al.^47^
**Shift to different setpoint**									
	7.8→6.8	CHO DUXB	Eg2-hFC	Batch	500 mL shake flask, shift at day 4	↑FA2G0, ↑FA2G1, ↑FA2G2	Not reported FI, no effect on GI, SI	✓	Aghamohseni et al.^27^
	7.05→6.75, 6.90	CHO DG44	IgM	Batch	1.5 L bioreactor, shift at day 2	Not reported	No effect on FI, ↑SI	Taken from the article	Hennicke et al.^29^
	7.0→6.8, 6.9	CHO	IgG1	Fed-batch	50 mL culture, shift at day 5	No effect on FA2G0, FA2G1, FA2G2	Not reported	✓	Yang & Ierapetritou^40^
	7.15→6.7–7.0	CHO S	IgG	Fed-batch	Bioreactor, shift at day 7	↓FA2G0, ↑FA2G1, ↑FA2G2	Not reported	✓	Villiger et al.^48^
	6.95→6.75	CHO GS	IgG1	Fed-batch	2 L bioreactor, shift at day 6	↑FA2G0, ↓FA2G1, ↓FA2G2	Not reported SI, No effect on FI, ↓GI	✓	Xie et al.^34^
	7.15→6.85	CHO	IgG1	Perfusion	15 L bioreactor, shift at day 9	↓FA2G0, ↑FA2G1, ↑FA2G2	Not reported	✓	Zheng et al.^49^
**Oscillatory pH**									
	6.9↔7.3	CHO GS	IgG1	Fed-batch	15 mL bioreactor, oscilation every 15, 30, 60 min	↑FA2G0	↓(FA2G1+FA2G2)	Taken from the article	Zakrzewski et al.^50^

Abbreviations: DoS, degree of sialylation [mol sialic acid/mol EPO-Fc]; FI, fucosylation index; GI, galactosylation index; SAC, sialic acid content [%]; SI, sialylation index.

**Table 3.** Reported effect of culture dissolved oxygen and CO_2_ partial pressure manipulations on glycan distribution, including glycoform distribution and glycan indices, of therapeutic IgG-related products in CHO cell systems.

Type of manipulation		Cell line	Product	Culture mode	Culture timeline	Reported effect		Suitable for calculating glycan indices	Reference
						On glycoform distribution	On net content		
***Dissolved oxygen***									
**Constant higher setpoint**									
	40% DOT	CHO	mAb	Batch	3 L bioreactor	Not reported	No effect on FI, GI, SI	Taken from the article	Brunner et al.^28^
	50, 70, 90, 100% DOT	CHO DUXB	EPO-Fc	Batch	5 L bioreactor	Not reported	⇅DoS	✗	Trummer et al.^36^
	100% DOT	CHO-K1	IgG1	Fed-batch	2.2 L bioreactor	⇅FA2G1, ⇅A1G0, ⇅M3	Not reported	✗	St. Amand et al.^41^
	50% DOT	CHO GS	IgG1	Fed-batch	15 mL bioreactor	↓FA2G0	↑(FA2G1+FA2G2)	Taken from the article	Zakrzewski et al.^50^
**Constant lower setpoint**									
	10% DOT	CHO	mAb	Batch	3 L bioreactor	Not reported	No effect on FI, GI, SI	Taken from the article	Brunner et al.^28^
	10% DOT	CHO-K1	IgG1	Fed-batch	2.2 L bioreactor	⇅FA2G1, ⇅A1G0, ⇅M3	Not reported	✗	St. Amand et al.^41^
	10% DOT	CHO DUXB	EPO-Fc	Batch	5 L bioreactor	Not reported	↓DoS	✗	Trummer et al.^36^
	10% DOT	CHO GS	mAb	Batch	250 mL bioreactor	Not reported	No effect on FI, ↑GI	Taken from the article	Madabhushi et al.^38^
	15% DOT	CHO DG44	IgG-fusion	Fed-batch	5 L bioreactor	Not reported	↓SAC	✗	Jing et al.^44^
	10% DOT	CHO GS	IgG1	Fed-batch	15 mL bioreactor	↓FA2G0	↑(FA2G1+FA2G2)	Taken from the article	Zakrzewski et al.^50^
**Oscillatory DOT**									
	8↔37% DOT	CHO GS	IgG1	Fed-batch	15 mL bioreactor, oscilation every 15, 30, 60 min	↑FA2G0	↓(FA2G1+FA2G2)	Taken from the article	Zakrzewski et al.^50^
***Other***									
**Antioxidant addition**									
	100 μM balcalein	CHO DG44	mAb	Batch	125 mL shake flask, balcalein addition at day 3	↓FA2G0, ↑FA2G1, no effect on FA2G2	Not reported	✓	Ha et al.^51^
	15 mM S-sulfocysteine	CHO	mAb	Fed-batch	1.2 L bioreactor, S-sulfocysteine feed since day 3	↓FA2G0, ↑FA2G1	Not reported	✓	Hecklau et al.^52^
**Higher *p*CO2 setpoint**									
	12.5, 20% *p*CO2	CHO	mAb	Batch	3 L bioreactor	Not reported	No effect on FI, ↓GI, ↓SI	Taken from the article	Brunner et al.^28^

Abbreviations: DoS, degree of sialylation [mol sialic acid/mol EPO-Fc]; FI, fucosylation index; GI, galactosylation index; SAC, sialic acid content [%]; SI, sialylation index.

**Table 4.** Reported effect of osmolality manipulations on glycan distribution, including glycoform distribution and glycan indices, of therapeutic IgG-related products in CHO cell systems.

Type of manipulation		Cell line	Product	Culture mode		Culture timeline	Reported effect				Suitable for calculating glycan indices		Reference
							On glycoform distribution		On net content				
***Osmolality***													
**Higher osmolality**													
	+100 mOsm/kg	CHO	DUKX-Fc		Batch	125 mL shake flask, 62.5 mM NaCl addition since day 0	↑FA2G0, ↓FA2G1, ↓FA2G2	Not reported		✓		Lee et al.^53^	
	+100, 200 mOsm/kg	CHO GS	mAb		Batch	250 mL bioreactor, NaCl addition since day 1	↑A2G0, ↓FA2G0, ↓FA2G1	No effect on FI, ↓GI		✓		Madabhushi et al.^38^	
	+60, 120, 180 mOsm/kg	CHO-K1	IgG		Fed-batch	250 mL shake flask, NaCl addition since day 8	Not reported	↑FI, ↓GI		Taken from the article		Qin et al.^33^	
	+75, 170 mOsm/kg	CHO GS	IgG1		Fed-batch	250 mL bioreactor, NaCl addition since day 0	Not reported	Not reported FI, SI, ↓GI		Taken from the article		Jiang et al.^47^	
	+370, 420, 470 mOsm/kg	CHO GS	IgG4		Fed-batch	250 mL shake flask, NaCl addition since day 0	↑FA2G0, ↓FA2G1, ↓FA2G2	Not reported		✓		Alhuthali et al.^54^	
	+410, 460, 500 mOsm/kg	CHO GS	IgG4		Fed-batch	250 mL shake flask, feed addition since day 0	⇅FA2G0, ↓FA2G1, ↓FA2G2	Not reported		✓		Alhuthali et al.^54^	
	+60, 120, 180 mOsm/kg	CHO-K1	IgG		Perfusion	250 mL shake flask, NaCl addition since day 6	Not reported	↑FI, ↓GI		Taken from the article		Qin et al.^33^	

Abbreviations: FI, fucosylation index; GI, galactosylation index; SAC, sialic acid content [%]; SI, sialylation index.

**Table 5.** Reported effect of chemical additives supplementation on glycan distribution, including glycoform distribution and glycan indices, of therapeutic IgG-related products in CHO cell systems.

Type of manipulation		Cell line	Product	Culture mode	Culture timeline	Reported effect		Suitable for calculating glycan indices	Reference
						On glycoform distribution	On net content		
***Chemical additives***									
**HDAC inhibitor addition**									
	5 mM NaBu	CHO	mAb	Batch	100 mL spinner flask	Not reported	No effect on SAC	✗	Chen et al.^37^
	2 mM NaBu	CHO-K1	IgG3	Batch	24-well plate, NaBu addition since day 1	Not reported	↓SAC	✗	Mimura et al.^55^
	3 mM NaBu	CHO DUXB	Fc-fusion	Batch	250 mL shake flask, NaBu addition since day 3	Not reported	↓SAC	✗	Lee et al.^56^
	0.2, 1, 2, 4 mM NaBu	CHO DG44	mAb	Batch	250 mL shake flask, NaBu addition since day 0	↑FA2G0, ↓FA2G1, ↓FA2G2	Not reported	✓	Hong et al.^57^
	5 mM NaBu	CHO GS	mAb	Batch	250 mL bioreactor, NaBu addition since day 4	No effect on glycoforms	No effect on FI, GI	✓	Madabhushi et al.^38^
	3.5 mM valproic acid	CHO	mAb	Fed-batch	5 L bioreactor, valproic acid addition since day 0	↑FA2G0, ↑FA2G1, ↑FA2G2	Not reported	✓	Yang et al.^58^
	1.5 mM valeric acid	CHO GS	IgG1	Fed-batch	2 L bioreactor, valeric acid addition since day 0	↓FA2G0, ↑FA2G1, no effect on FA2G2	Not reported	✓	Park et al.^59^
**qP-enhancing chemical addition**									
	10 mM LiCl	CHO	DUKX-Fc	Batch	125 mL shake flask, 10 mM LiCl addition since day 0	Not reported	↓SAC	✗	Ha et al.^60^
	5 μM CDK4/6 inhibitor	CHO	mAb	Batch	24-well plate, CDK4/6 inhibitor addition at day 1	↓FA2G0, ↓FA2G1, ↓FA2G2	Not reported	✓	Du et al.^61^
	50 μM BIX and 1%v/v DMSO	CHO GS	IgG1	Fed-batch	1.5 mL bioreactor, BIX and DMSO addition at day 3	↓FA2G0, ↑FA2G1, ↑FA2G2	Not reported	✓	Ha et al.^62^
	5 μM foskolin	CHO GS	IgG1	Fed-batch	125 mL shake flask, foskolin addition at day 3	↓FA2G0, ↑FA2G1, no effect on FA2G2	Not reported	✓	Yoon et al.^63^

Abbreviations: FI, fucosylation index; GI, galactosylation index; SAC, sialic acid content [%]; SI, sialylation index.

### Overview of glycan indices

**Figure 3.**
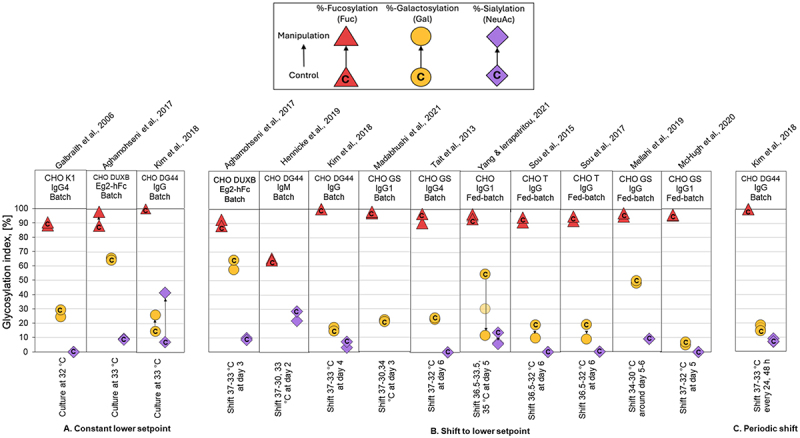
Comparison of glycoforms distribution changes reported for experiments using temperature manipulation to affect the N-linked glycosylation pattern of recombinant IgG-producing CHO cell systems. The %-change in glycoforms distribution is shown as a displacement from a starting control distribution, indicated with a ‘C’ within the respective symbol, to a final distribution following manipulation, as per the directional arrow between the points. Control: culture at 37°C; manipulation: lower setpoint (A), temperature shift (B) and periodic shift (C). The GIx % for galactosylation (yellow circle), sialylation (violet diamond), and fucosylation (red triangle) are represented by their respective monosaccharide symbols.

**Figure 4.**
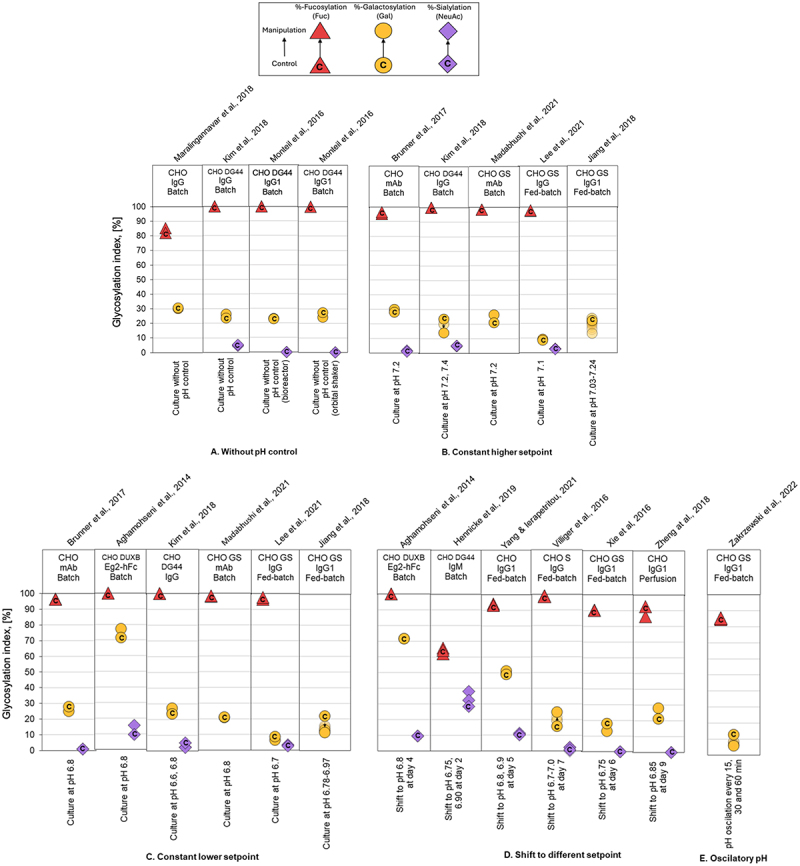
Comparison of glycoforms distribution changes reported for experiments using pH manipulation to affect the N-linked glycosylation pattern of recombinant IgG-producing CHO cell systems. The %-change in glycoforms distribution is shown as a displacement from a starting control distribution, indicated with a ‘C’ within the respective symbol, to a final distribution following manipulation, as per the directional arrow between the points. Control: culture at pH 7.0; manipulation: without pH control (A), constant higher setpoint (B), constant lower setpoint (C), shift to different setpoint (D), and oscillatory pH (E). The GIx % for galactosylation (yellow circle), sialylation (violet diamond), and fucosylation (red triangle) are represented by their respective monosaccharide symbols.

**Figure 5.**
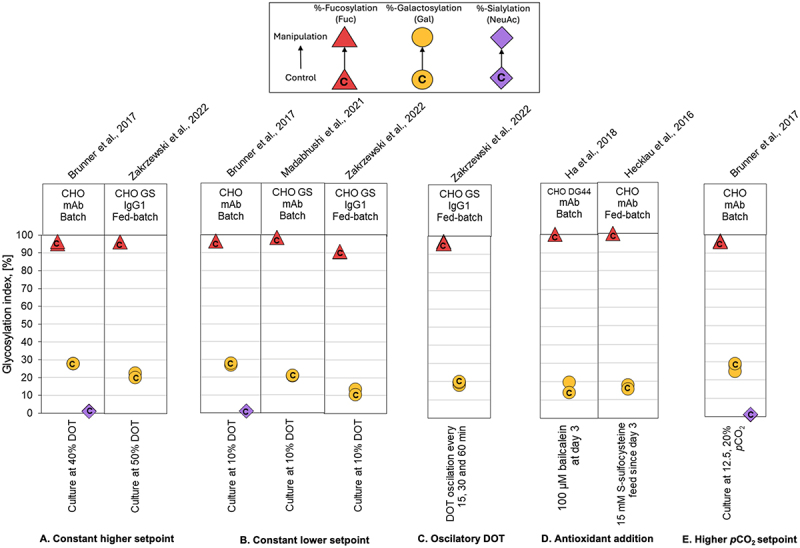
Comparison of glycoforms distribution changes reported for experiments using DOT/antioxidants/*p*CO_2_ manipulation to affect the N-linked glycosylation pattern of recombinant IgG-producing CHO cell systems. The %-change in glycoforms distribution is shown as a displacement from a starting control distribution, indicated with a ‘C’ within the respective symbol, to a final distribution following manipulation, as per the directional arrow between the points. Control: culture at 25–30% DOT/culture at 5% *p*CO_2_; manipulation: constant higher DOT (A), constant lower DOT (B), and oscillatory DOT (C), antioxidant addition (D) and higher *p*CO_2_ setpoint (E). The GIx % for galactosylation (yellow circle), sialylation (violet diamond), and fucosylation (red triangle) are represented by their respective monosaccharide symbols.

**Figure 6.**
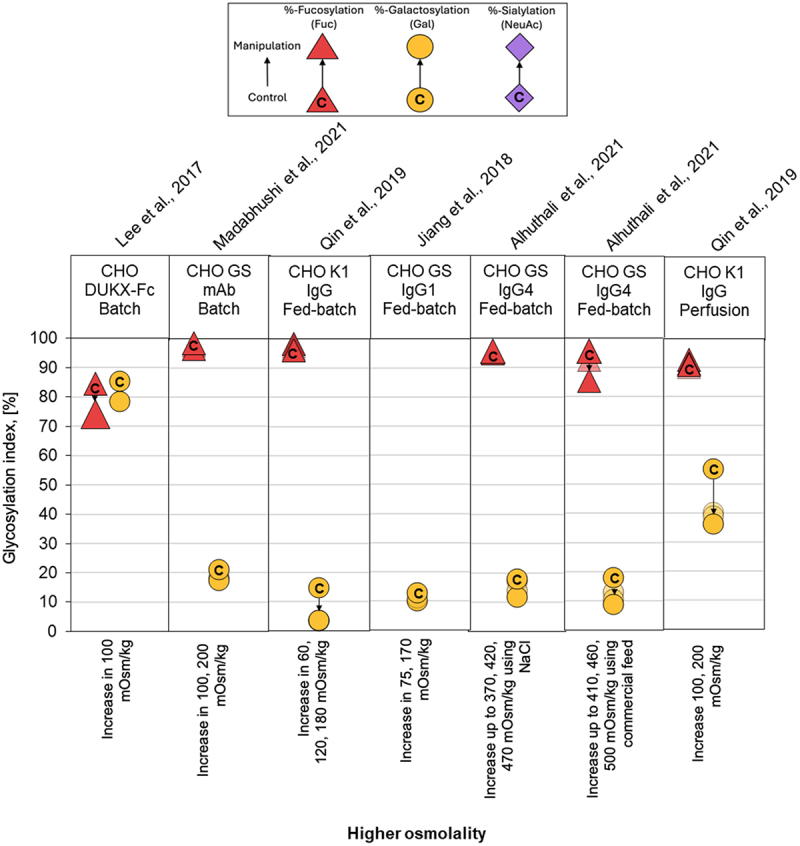
Comparison of glycoforms distribution changes reported for experiments using osmolality manipulation to affect the N-linked glycosylation pattern of recombinant IgG-producing CHO cell systems. The %-change in glycoforms distribution is shown as a displacement from a starting control distribution, indicated with a ‘C’ within the respective symbol, to a final distribution following manipulation, as per the directional arrow between the points. Control: natural osmolality of the culture medium; manipulation: higher osmolality. Those cases where the osmolality was not reported and only the perturbation was described are indicated in the form *increase in*; on the contrary, when it was measured point by point are indicated in the form *increase up to*. The GIx % for galactosylation (yellow circle), sialylation (violet diamond), and fucosylation (red triangle) are represented by their respective monosaccharide symbols.

**Figure 7.**
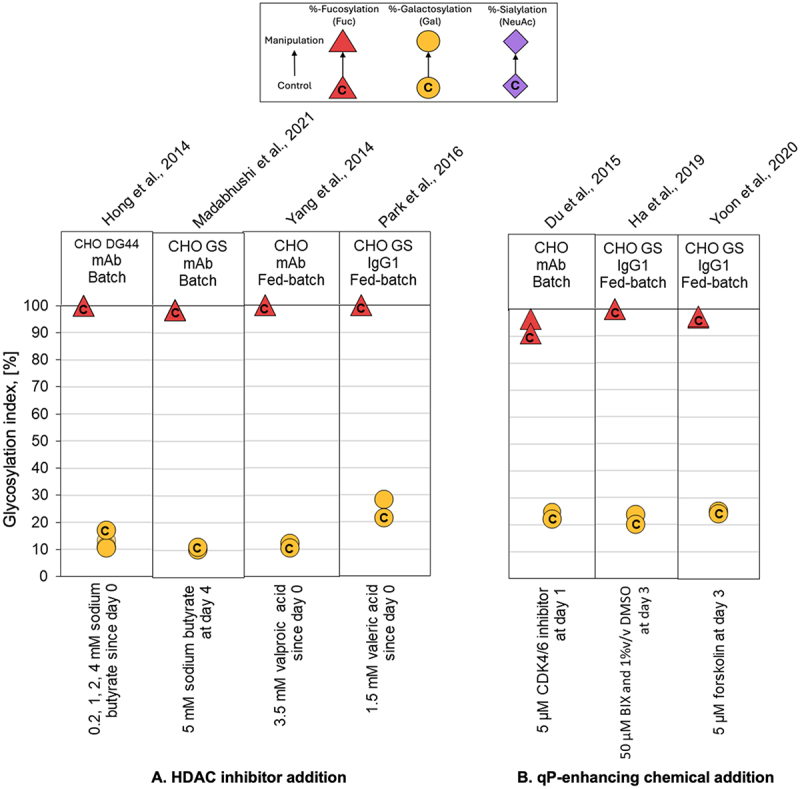
Comparison of glycoforms distribution changes reported for experiments using supplementation of chemical compounds to affect the N-linked glycosylation pattern of recombinant IgG-producing CHO cell systems. The %-change in glycoforms distribution is shown as a displacement from a starting control distribution, indicated with a ‘C’ within the respective symbol, to a final distribution following manipulation, as per the directional arrow between the points. Control: culture medium without supplementation; manipulation: HDAC inhibitor addition (A) and qP-enhancing chemical addition (B). The GIx % for galactosylation (yellow circle), sialylation (violet diamond), and fucosylation (red triangle) are represented by their respective monosaccharide symbols.

This study aimed to quantify and compare the impact of PPs on the Fc N-glycosylation patterns in IgG-type mAbs produced in CHO systems. We systematically reviewed peer-reviewed studies reporting glycan distributions under defined process conditions. We standardized the heterogeneous glycosylation data by converting glycoform abundances to GIx using methodology of Blondeel & Aucoin.^26^ Our analysis of FI, GI, and SI (Figures 3–7) revealed that, in most studies, mAb glycosylation fell within typical ranges of FI > 85%, GI at 10–30% range and SI < 10%.^64^

We observed that glycosylation profiles demonstrated a high degree of stability across environmental culture conditions, CHO cell lines, culture modalities, and the specific IgG produced. Most PP modifications, including pH, DO, partial pressure of CO_2,_ and chemical additives supplementation, led to less than 10% deviation the GIx value (ΔGIx), even in some cases changes below 1.5% (Figures 3–7; Supplementary Appendix). These results are consistent with previously observations.^41^ In a series of cases manipulating temperature or osmolality had a noticeable impact on GI and FI. These cases are relevant because even a small deviation in FI can have a significant impact on biological activity.^65^ Impacts of similar magnitude were observed for the summative glycan indices (Supplementary Appendix), confirming the trends observed for GIx.

In our analysis, variability among the reviewed studies further limited the interpretation of results. This variability arises because each work generated data from systems that differed in design and execution, making the conclusions specific to each individual setup. In some cases, we observed batch-to-batch differences under the same condition (Figure 7B-C),^47^ or inconsistent/contradictory glycan outcomes under similar or comparable process optimization strategies.^28^ These findings indicate that PPs alone are insufficient to reliably direct glycosylation toward a desired profile. To achieve more desirable glycosylation patterns, complementary strategies such as culture media optimization (e.g., nutrients, trace metals, amino acids, hormones) are required.^41,66^ Blondeel & Aucoin^26^ systematically reviewed the impact of culture media supplementation on glycosylation using the GIx approach; the reported supplementation strategies with significant ΔGIx serve as a positive control to the results presented in our work. Galactose supplementation combined with manganese increased the GI by more than 20% in multiple IgG-producing CHO systems. These cases demonstrate the precision of the GIx methodology, which is capable of measuring with high sensitivity cases involving major changes in glycan net content, as well when the ΔGIx is limited or absent.

### Manipulation of temperature

Modulating culture temperature during the production phase is a widely reported strategy that is routinely implemented in the biopharmaceutical industry. We found that the most reported approaches are: (1) a temperature downshift (from 37°C to 30–35°C) at later culture stage (Figure 3B), and (2) growing cells at low temperature from the start, sometimes including adaptation at early passages (Figure 3A). Less common was the use of periodic temperature shifts (Figure 3C).

Our analysis indicated that lowering culture temperature may affect the net content of galactose and fucose, thereby impacting biological activity. Of nine cases studies, one showed ≥ 10% increase in GIx,^23^ three had ≥10% reductions in GIx,^24,43^ with only one study presenting a large decrease.^40^ Most other cases reported minimal change in galactose net content, and sialylation generally remained unaltered.

A plausible explanation for the impact of temperature on the GIx lies in the multiple cellular responses triggered by temperature reduction. Lowering culture temperature can decrease cell metabolism, cell proliferation, and expression of nucleotide sugar donor transporters and glycosyltransferases.^38,40,42^ At the same time, it also slows secretion of endogenous proteins other than the recombinant mAb,^67^ possibly reducing competition for cellular resources. However, the increase in specific productivity (qP) typically observed at reduced temperatures shortens the residence time of the mAb in the secretory pathway, particularly under conditions of limited glycan precursor availability. Therefore, the use of mild hypothermia can sometimes lead to the accumulation of incompletely processed glycans (Figure 3B). Further studies are required to confirm the relative contribution of these mechanisms.

### Manipulation of pH

pH is a critical PP during cell culture, commonly maintained at 7.0 using CO_2_ sparging or base addition (e.g., sodium hydroxide, sodium carbonate, sodium bicarbonate) as control tools.^68^ Although several studies have investigated the pH effect on glycosylation,^46^ we found no consistent response to shifting pH away from baseline (Figure 4). In some cases, pH > 7.0 led to increased glycan heterogeneity, whereas in others, it decreased.^47^ Lowering pH below 7.0 yielded a 2.32% increase in GI and a 0.7% decrease in SI, whereas pH > 7.0 had negligible impact. However, these contradictory observations, even for the same product and cell line, complicate efforts to establish a universal framework describing pH effects on glycosylation. In cases where there was a change in GIx, it was mainly evident in galactose, with a ∆GIx of less than 10%.

Mechanistically, pH effects are less studied than those of temperature. At lower pH values (e.g., 6.7), excess extracellular protons can increase cytosolic and, consequently, luminal pH in the endoplasmic reticulum (ER) and Golgi, disrupting the Golgi pH gradient. Increased ER-to-Golgi vesicular trafficking might also shorten the residence time of proteins in the secretory pathway, causing early release of proteins with partially processed glycans.^46^ This mechanism contrasts with the effects often associated with low temperature.

### Manipulation of dissolved oxygen and carbon dioxide partial pressure

DO is another critical PP, commonly monitored as dissolved oxygen tension (DOT) with setpoints around 20–50%. Oxygen transfer depends on gas flow rate, oxygen partial pressure in the inlet gas, bubble size, agitation rate, and impelled design.^69^ Low DO levels can induce hypoxia, whereas high DO levels can cause oxidative stress and reactive oxygen species production. Both conditions can impair cell growth, cell survival, mAb production, and product quality.^70^ Only a few studies have systematically investigated the effect of varying DO on glycosylation,^28,38,50^ with most reporting minimal changes in GIx (∆GIx < 1.0%) (Figure 5A-C). There are no studies with a mechanistic oxygen-dependant approach on glycosylation for IgG-expressing CHO cell lines; therefore, there is significant room for a better understanding of this phenomenon.

To mitigate oxidative stress under high DO conditions, researchers have supplemented the culture with antioxidants, aiming to reduce reactive oxygen species generation and preventing oxidative damage. Studies supplementing baicalein^51^ and S-sulfocysteine,^52^ an L-cysteine analogue with antioxidant properties, reported no significant variations of glycosylation indices when DO was controlled at 50% air saturation (Figure 5D). For these cases, the galactose content was discreetly increased with a ∆GIx of 2–5%. However, these studies did not present challenge experiments, where antioxidant supplementation could mitigate high DO conditions.

*p*CO_2_ also influences culture performance, primarily through its role in pH control. Typically, mammalian cell culture is set at 5% CO_2_, and adjusted as needed to maintain pH.^71^ Our understanding of the impact of CO_2_ on mAb glycosylation is limited, in part because its variations indirectly affect other parameters such as pH.^28^ Studies show that increasing *p*CO_2_ above 5% marginally reduces GI (<∆GIx 5%) without affecting fucosylation or sialylation (Figure 5E).

### Manipulation of osmolality

Osmolality is critical for cell growth, viability and productivity,^71^ being often increased in cultures enriched by feeds or with accumulated by-products. Typical osmolality values in mammalian cell culture range from 280 to 320 mOsm/kg.^35^ However, hyperosmolality induced by adding NaCl can enhance cell-qP.^72^ Various process controls can modulate biomass formation and product yield, raising the key question of whether these parameters simultaneously influence protein glycosylation. In the studies analyzed, increasing osmolality consistently decreased GI by about ∆GIx of 10% (Figure 6), and a decrease in FI ∆GIx 2–10%. This significant impact on the content of galactose and fucose would have a strong effect on the mAb therapeutic functionality. However, the variability in baseline osmolality among studies complicates direct comparisons.

Mechanistically, elevated osmolality downregulates genes involved in glycosylation, including those encoding nucleotide sugar transporters, key glycosyltransferases, thereby limiting substrate and cofactor availability and resulting in less processed glycans.^38,53^ This phenomenon raises questions about intensified fed-batch processes, where osmolality might drastically fluctuate and alter glycosylation patterns. Consequently, implementing osmolality-elevating strategies to enhance productivity requires concurrent measures to maintain product quality throughout the culture.

### Supplementation of productivity-enhancing molecules

Several productivity-enhancing molecules are routinely used in mammalian cell cultures to increase product yields and specific cell productivity. These include chemicals that induce, either independently or in combination, cell cycle arrest (e.g., cyclin-dependent kinase inhibitors),^73^ chemical chaperones,^74^ ER stress inhibitors,^70^ and histone deacetylase inhibitors (HDAC) such as NaBu or valeric acid.^38^ Our analysis shows that respect the galactosylation content, in half of the cases there is an effect around ∆GIx 6%, with minor effects ∆GIx < 1.5% for the rest, and do not affect fucosylation (Figure 7). NaBu is known to alter the expression of several genes in the glycosylation pathway, including the manganese transporter gene (slc39a8), a key cofactor in the glycosyltransferase reaction,^38^ but the magnitude of this effect is insufficient to significantly change mAb glycosylation. Valproic and valeric acid molecular mechanisms affecting glycosylation remain unresolved, though they are expected to be similar to those proposed for NaBu.^58,59^

For example, CDK4/6 inhibitors induced G0/G1 arrest in CHO cells and increased galactose and fucose occupancy (<∆GIx 2–5%)^61^ (Figure 7B). Combining dimethyl sulfoxide (DMSO) with BiP inducer X (BIX) provided chemical chaperoning benefits while mitigating DMSO-induced ER stress, leading to enhanced B4galt expression and improved glycan occupancy (<∆GIx 5% for GI).^62^ Similarly, forskolin supplementation increased qP via G0/G1 arrest and Bcl-2 gene inhibition, and although the authors reported enhanced terminal galactosylation,^63^ the effect was minimal when assessed using the GIx approach (Figure 7B). These effects are highly process-dependent and require further investigation to clarify the molecular mechanisms linking chemical supplementation to glycan outcomes.

From an industrial perspective, manufacturers tend to avoid such supplements unless they offer substantial productivity gains because of their high cost at large scale, potential downstream removal challenge, and the risk of residual impurities. As cell line development and bioprocess optimization continue to advance, the need for these supplements has diminished, limiting their use in commercial settings.

The effect of the addition of chemical compounds on mAb-producing CHO cultures proved to be highly process-dependent. Additional work is needed to fully understand the molecular mechanisms that relate this strategy to the glycan analyses obtained.

## Discussion

This study aimed to provide a clear approach to compare how PPs changes affect mAb glycosylation. The literature reports glycan data in many formats, which makes comparison between studies complicated, slowing translation to manufacturing practices. To address this, we used GIx as common quantitative metric. Although previous studies have explored the impact of PPs on the glycosylation of mAb-related proteins, this is the first to apply a quantitative methodology to systematically evaluate these effects. The GIx calculation was used as a standardization tool for glycan reporting,^26^ providing a framework for comprehensive understanding and enabling objective comparisons of PP effects across different studies. GIx enabled us to standardize data from different cell lines, media, and operating conditions on the same metric. This approach also allows to patterns in previously published data sets to be identified. Overall, we found that the GIx enabled comparison of PP interventions across different studies. In this way we revealed the robustness of glycosylation in response to changes of several process operations, but also identified key cases where activity could be severely affected.

We found that GIx values appear relatively stable under the different PP considered in this analysis. For instance, galactosylation only varied modestly across studies (Figures 3–7). Typical process interventions, such as pH set-points, temperature shifts, DO, osmolality control or small-molecule supplementation, tended to yield small and, sometimes, inconsistent changes in glycoforms. This stability might be explained by two factors. The first is associated with the narrow operation windows for process interventions (e.g., pH ~ 6.7–7.4; T 30–38 °C).^71^ The second is biological. Process interventions must overcome several buffered layers, including the plasma membrane, intracellular trafficking, ER/Golgi enzyme networks, before affecting glycan processing.^75^ Therefore, changes in other parameters (often cell growth or productivity) are more likely to be observed before mAb glycosylation. These data suggest that to move GIx in a significant manner, process interventions need to be of a magnitude that can affect multiple homeostatic controls at once. This may explain why isolated interventions of pH, DO, *p*CO_2_ or chemical additives supplementation often do little in altering glycosylation.

Temperature and osmolality exert global effects across cellular compartments and membranes, thereby influencing net fucosylation and galactosylation. In those cases, even modest shifts in Fc glycosylation can translate into meaningful changes in mAb function. Galactosylation and fucosylation both influence effector functions through ADCC, but afucosylation plays the dominant role. Even small reductions in core fucose markedly enhance FcγRIIIa binding and in vitro ADCC.^14,65^ Accordingly, process-induced changes in fucosylation on the order of 1–10%, such as those associated with temperature or osmolality shifts, can be biologically and therapeutically relevant, despite appearing minor at the GIx level.

GIx, used well, functions as sensible high-level indicator. A previous study has extensively reviewed different interventions and the consequences of glycan distribution^35^ However, the approach taken was mainly qualitative, lacking a numerical perspective to the glycan variations. GIx improved on that by reflecting net sugar occupancy and linking process conditions to attributes that matter for activity. This approach not only reaffirmed known patterns but also yielded novel insights, even from previously published glycan data. For instance, culture conditions identified to drive shifts in complex-type glycoforms (based solely on a Glycan Distribution approach)^27,30,38,46,63^ were reinterpreted through the GIx framework. In many such cases, the net changes in sugar occupancy proved marginal – often less than 5%—revealing a more limited effect assigned to PP. Also, GIx can compress distributions. For instance, galactose index can mask a redistribution between G1F and G2F that is relevant for bioactivity. Our practical recommendation is to report both, the full glycan distribution so the mechanism is visible and the corresponding GIx for inter-study comparison. This dual reporting is simple to implement and may contribute to lower the barrier to cumulative evidence.

What qualifies as a “good” glycosylation profile depends on the expected functionality of the specific mAb, which complicate general claims. For cytotoxic IgG1, lower core fucose generally improve FcγRIIIa binding and ADCC, while increased galactosylation can enhance C1q and CDC.^76^ When effector silencing is preferred (common for IgG4 or blocking IgG1), human-like, core-fucosylated complex glycans with low high-mannose and some sialylation tend to attenuate effector function and may support longer half-life.^77^ GIx can be mapped to these targeted functionalities, providing process development teams a quantitative way to a specific “good” profile for a given product rather than in the abstract.

From a regulatory perspective, the magnitude of the glycosylation changes we observed must be interpreted in the Quality by Design (QbD) framework, ideally in a case-by-cases basis. Regulatory agencies such the US Food and Drug Administration and European Medicines Agency emphasize that the impact of a CQA on safety and efficacy should be evaluated using a risk assessment and expressed relative to a change in clinical potency compared the reference standard.^78^ Case studies for biosimilars like Enbrel® (an IgG1-based fusion protein) and Remicade® (a mAb) demonstrated batch-to-batch differences exceeding ten percentage points in glycoform composition and total sugar content, yet without detectable effects on clinical performance.^79,80^ The process-induced glycan variations we reported here fall within or below these ranges, suggesting that typical PP adjustments are unlikely to impact biological efficacy meaningfully. These findings underscore the importance of quantitatively mapping PPs to glycan outcomes as part of defining a robust design space, a cornerstone of regulatory expectations under QbD. Such quantitative understanding enables manufacturers to rationally explore operating ranges that maintain product quality while supporting process flexibility and innovation.

This work has limitations. We restricted the scope of analysis to CHO-cell-derived mAbs, given their market and regulatory importance. Other expression systems and products were out of scope. The underlying studies vary in how they report glycans, often explore narrow or heterogeneous PP ranges, and rarely include mechanistic read-outs. Inter-study variability is high, scale-up tests are uncommon, and downstream consequences (e.g., additive carry-over, impurity profiles) are not routinely quantified.

Two additional sources of discrepancy further complicate cross-study comparisons. First, differences in culture systems (including bioreactor configuration, process control strategies, sensor technologies, and PP measurement approaches) can introduce variability.^71^ Parameters such as pH or *p*CO₂ are often assumed to be standard and directly comparable across literature. However, differences in control methods, calibration procedures, and sensor sensitivity can lead to non-negligible inconsistencies. Second, variability arises from the analytical methods used for glycan characterization. The use of diverse techniques, including chromatography-based methods, capillary electrophoresis, and multiple mass spectrometry platforms, can influence reported glycan distributions and indices, thereby affecting inter-study comparability^81^ (Supplementary Results).

A comprehensive evaluation of these methodological differences would require a dedicated analysis and falls outside the scope of the work presented here, which focuses on relating PPs to IgG glycosylation outcomes. Publication bias toward “positive” interventions may also shape the evidence base. Future work would benefit from standardized reporting templates (indices plus distributions), wider design spaces (formal DoE rather than one-factor changes), integration of multi-omics and flux analysis to link PP to nucleotide-sugar pools and enzyme expression, public deposition of raw glycan tables, and validation at pilot scales.

In conclusion our work proposed that quantitative and standardized analyses such as the GIx, are essential for objectively comparing how PPs influence mAb glycosylation. In most cases, environmental perturbations altered FI, GI, and SI by less than 10%, with many changes below 5% or even non-existent. However, in specific cases, particularly involving temperature and osmolality, shifts in fucosylation and galactosylation reached ranges that may have meaningful biological consequences. These process-related effects vary significantly across cell lines, products, and culture modes, and can differ on a batch-to-batch basis under identical nominal conditions. Consequently, the data suggests that adjusting PPs alone is an unreliable method for tailoring glycosylation patterns. A more rational approach is to maintain these parameters within appropriate control ranges (according to operation region or design space) and combine with targeted media modifications (nutrient, trace metal, or hormone supplementation) or genetic engineering. From the analysis carried out, it was evident that substantial work is still required to improve understanding of the mechanisms relating to the potential effect of PPs on glycosylation. Furthermore, the effect of PP on glycosylation has been scarcely studied for perfusion culture mode. By providing a comprehensive, quantitative understanding of how PPs affect this CQA, this work can inform better decision-making and foster improved production strategies for optimal mAb quality.

## Supplementary Material

Supplementary Appendix.docx

## Abbreviations

A: Antenarity
ADCC: Antibody-dependent cell-mediated cytotoxicity
Bcl-2: B-cell lymphoma 2
BIX: BiP inducer X
CDK4/6: Cyclin-dependent kinase 4/6
CHO: Chinese hamster ovary
CQA: Critical Quality Attribute
DMSO: Dimethyl sulfoxide
DO: Dissolved oxygen
DoS: Degree of sialylation
DOT: Dissolved oxygen tension
ER: Endoplasmic reticulum
Fab: Antigen binding fragment
Fc: Crystallisable fragment
F: Fucose occupancy
FI: Fucosylation index
Fuc: Fucose
G: Galactose occupancy
Gal: Galactose
GI: Galactosylation index
GIx: Glycan index
ΔGIx: Difference in glycan index
GlcNAc: N-acetylglucosamine
H: Heavy chain
HDAC: Histone deacetylase inhibitors
IgG: Immunoglobulin G
L: Light chain
mAb: Monoclonal antibody
Man: Mannose
M: Mannose occupancy
NaBu: Sodium butyrate
NeuAc: N-acetylneuraminic acid
*p*CO₂: Carbon dioxide partial pressure
PP: Process parameter
PTM: Post-translational modification
qP: Specific productivity
QbD: Quality by Design
S: NeuAc occupancy
SAC: Sialic acid content
SI: Sialylation index.
