# Supplementary material for: Impact of process parameters on IgG glycosylation in CHO systems: a comprehensive quantitative analysis
Source: MAbs. 2026 Mar 15;18(1):2643039. doi: 10.1080/19420862.2026.2643039 (PMC12990948; doi:10.1080/19420862.2026.2643039)
Supplement: Supplementary Appendix.docx [file KMAB_A_2643039_SM5049.docx]

**Impact of process parameters on IgG glycosylation in CHO systems: a comprehensive quantitative analysis**

Javier Bravo-Venegas^1,2,3^, Jose Rodriguez-Siza^3^, Mauricio Vergara^3,4^, Mauro Torres^1,2,5^, Alan Dickson^1,2^, Jorge R. Toledo^6^, María Carmen Molina^7^, Marcela A. Hermoso^8,9^, Julio Berríos^3,4^, Claudia Altamirano^3,4,10,11^

**Affiliations**: ^1^Department of Chemical Engineering, University of Manchester, Manchester, UK. ^2^Manchester Institute of Biotechnology, Faculty of Science and Engineering, University of Manchester, Manchester, UK. ^3^School of Biochemical Engineering, Faculty of Engineering, Pontificia Universidad Católica de Valparaíso, Av Brasil 2085, Valparaíso, Chile. ^4^Center for Interdisciplinary Research in Biomedicine, Biotechnology and Well-Being (CID3B). Pontificia Universidad Católica de Valparaíso, Valparaíso, Chile. Av. Brasil 2950, Valparaíso, Chile. ^5^Department of Chemical Engineering, Biochemical and Bioprocess Engineering Group, University of Manchester, Manchester, UK. ^6^Laboratorio de Biotecnología y Biofarmacia, Departamento de Fisiopatología, Facultad de Ciencias Biológicas, Universidad de Concepción, Concepción 4030000, Chile. ^7^Laboratorio de anticuerpos recombinates e inmuno-oncologia, Núcleo Interdisciplinario de Farmacología e Inmunología (NIFI), Instituto de Ciencias Biomédicas (ICBM), Facultad de Medicina, Universidad de Chile, Avda. Independencia 1027, Block I, 3er piso, Santiago, Chile. ^8^Immunology Programme, Faculty of Medicine, University of Chile, Santiago, Chile. ^9^Department of Gastroenterology and Hepatology, University of Groningen, Groningen, The Netherlands. ^10^Centro Regional de Estudio en Alimentos Saludables, R17A10001, Av. Universidad 330, Valparaíso, Chile. ^11^IMPACT, Center of Interventional Medicine for Precision and Advanced Cellular Therapy, Santiago, Chile.

**SUPPLEMENTARY APPENDIX**

**SUPPLEMENTARY METHODS**

*Example of glycan indices calculation*

To determine the Glycan Indices (GIx), glycoform distribution information was collected for each experimental article. As an example, part of the dataset reported by by St. Amand et al. (2016) for day 12 of the custom fractional factorial design of experiments is presented (Table S1). Fucosylation (FI), galactosylation (GI), and sialylation (SI) indices were calculated following the methodology described by Blondeel & Aucoin (2018). The equations reflect the percentage of occupied fucose, galactose, or sialic acid sites, based on coefficient assigned to each glycoform. For each moiety, the GIx was calculated according to the following equations:

|  | $GI=\frac{\sum_{g=1} g\sum_{i=1} G_{i}^{g}}{\sum_{g=0} \sum_{a=1} a\sum_{j=1} G_{j}^{g}}\times100$ | (1) |
| --- | --- | --- |
|  | $SI=\frac{\sum_{s=1} s\sum_{i=1} G_{i}^{s}}{\sum_{s=0} \sum_{g=1} g\sum_{j=1} G_{j}^{s}}\times100$ | (2) |
|  | $FI=\frac{\sum_{f=1} f\sum_{i=1} G_{i}^{f}}{\sum_{f=0} \sum_{j=1} G_{j}^{f}}\times100$ | (3) |

Where:

*G_i_^*^* is the portion of glycoforms with a particular moiety (g, s and f) and

*G_j_^*^* is the portion of glycoforms able to receive a particular moiety.

*g* is the number of terminal galactose moieties per glycan

*a* is the number of terminal GlcNAc moieties per glycan.

*s* is the number of terminal sialic acid moieties per glycan.

*f* is the binary number of core *α_(1_*_→_*_6_*_)_ fucosylation per glycan.

The change in a determined GIx between a control and a manipulation condition (ΔGIx) was considered as the absolute percentage difference. Additionally, the summative indices for each sugar moiety were calculated to evidence global changes in distributions. Note that in the case of fucose, the results were presented as a summative index for fucose, to continue the trend presented for the fucose index (FI). Naturally, the sum of afucosylates would correspond to the difference to complete 100%. The summative indices were calculated according to the following equations, which take into account all pertinent species with galactose, fucose or sialic acid motifs respectively:

|  | $Summ index galactose=\sum_{i=1} G_{i}^{g}$ | (4) |
| --- | --- | --- |
|  | $Summ index sialic acid=\sum_{i=1} G_{i}^{s}$ | (5) |
|  | $Summ index fucose=\sum_{i=1} G_{i}^{f}$ | (6) |

Where:

*G_i_^*^* is the portion of glycoforms with a particular moiety (g, s and f).

ΔSummative index was defined as the absolute percentage difference between control and manipulated conditions. For studies that reported only glycan indices without providing full glycan distributions, the published glycan indices were used as reported by the authors. In these cases, summative indices could not be calculated.

Table S1. Glycan index (GIx), glycan indices difference, (∆GIx) and summative indices calculation for run 1 and 5 according to the glycoform distribution described by St. Amand et al. (2016).

| **Glycoform, [%]** | Coefficient | | | | St. Amand et al. (2016) | |  | |
| --- | --- | --- | --- | --- | --- | --- | --- | --- |
|  | a | f | g | s | Run 1 | Run 5 |  | |
| A1G0 | 1 | 0 | 0 | 0 | 9.56 | 10.24 |  | |
| A2G0 | 2 | 0 | 0 | 0 | 1.33 | 2.73 |  | |
| FA1G0 | 1 | 1 | 0 | 0 | 8.41 | 6.52 |  | |
| FA2G0 | 2 | 1 | 0 | 0 | 50.45 | 35.52 |  | |
| A1G1 | 1 | 0 | 1 | 0 | 1.76 | 2.95 |  | |
| A2G1 | 2 | 0 | 1 | 0 | 5.09 | 3.87 |  | |
| FA1G1 | 1 | 1 | 1 | 0 | 2.69 | 3.66 |  | |
| FA2G1 | 2 | 1 | 1 | 0 | 2.72 | 4.35 |  | |
| A2G2 | 2 | 0 | 2 | 0 | 0.4 | 0.63 |  | |
| FA2G2 | 2 | 1 | 2 | 0 | 0 | 0.41 |  | |
| A2G2S1 | 2 | 0 | 2 | 1 | 0 | 0 |  | |
| FA1G1S1 | 1 | 1 | 1 | 1 | 0 | 0.23 |  | |
| FA2G1S1 | 2 | 1 | 1 | 1 | 0 | 0.17 |  | |
| FA2G2S1 | 2 | 1 | 2 | 1 | 0 | 0.13 |  | |
|  |  |  |  | **Glycan Indices, [%]** | | | | **Difference (∆GIx), [%]** |
|  |  |  |  | FI | 77.99 | 71.4 | -6.58 | |
|  |  |  |  | GI | 9.17 | 14.74 | 5.57 | |
|  |  |  |  | SI | 0 | 3.02 | 3.02 | |
|  |  |  |  | **Summative indices, [%]** | | | | **Difference (∆Summ index), [%]** |
|  |  |  |  | Fucose | 64.27 | 50.99 | -13.28 | |
|  |  |  |  | Galactose | 12.66 | 16.4 | 3.74 | |
|  |  |  |  | Sialic acid | 0 | 0.53 | 0.53 | |

**SUPPLEMENTARY RESULTS**

*Overview of glycan indices (GIx), glycan indices difference (∆GIx) and summative indices*

To facilitate analysis of the results, the data presented in Figures 3–7 are provided in tabular form, including the difference between the control and manipulated conditions. The summative indices for each case, along with the analytical method used to perform the glycan analysis, are also reported.

Table S2. Calculated impact of culture temperature manipulations on glycan indices (GIx), glycan indices difference (∆GIx) and summative indices, and described method for glycan analysis of therapeutic IgG-related products in CHO cell systems.

| Kind of manipulation | | Sugar motive | GIx control, [%] | GIx manipulation, [%] | ∆GIx, [%] | Summative index control, [%] | Summative index manipulation, [%] | ∆Summative index, [%] | Method of glycan analysis | Reference |
| --- | --- | --- | --- | --- | --- | --- | --- | --- | --- | --- |
| ***Temperature*** | | | | | | | | | | |
| **Constant lower setpoint** | | | | | | | | | | |
|  | 32 °C | Fucose | 90.36 | 88.3 | -2.06 | 90.36 | 88.3 | -2.06 | MALDI-MS | Galbraith et al. (2006) |
|  |  | Galactose | 29.19 | 24.27 | -4.92 | 47.72 | 39.18 | -8.54 |  |  |
|  |  | Sialic acid | 0 | 0 | 0 | 0 | 0 | 0 |  |  |
|  | 33 °C | Fucose | 88.31 | 98.11 | 9.8 | 88.06 | 98.3 | 10.24 | 2-AB labelling, HILIC | Aghamohseni et al. (2017) |
|  |  | Galactose | 64.2 | 65.72 | 1.52 | 80.8 | 84.57 | 3.77 |  |  |
|  |  | Sialic acid | 8.56 | 9.29 | 0.73 | 9.78 | 10.27 | 0.49 |  |  |
|  | 33 °C | Fucose | 100 | 100 | 0 | 103 | 102 | -1 | 2-AA labelling, HPLC | Kim et al. (2018) |
|  |  | Galactose | 14.56 | 25.98 | 11.42 | 25 | 37 | 12 |  |  |
|  |  | Sialic acid | 7.14 | 41.38 | 34.24 | 1 | 12 | 11 |  |  |
| **Shift to lower setpoint** | | | | | | | | | | |
|  | 37➝33 °C | Fucose | 88.31 | 92.71 | 4.4 | 88.06 | 93.89 | 5.83 | 2-AB labelling, HILIC | Aghamohseni et al. (2017) |
|  |  | Galactose | 64.2 | 57.53 | -6.67 | 80.8 | 74.08 | -6.72 |  |  |
|  |  | Sialic acid | 8.56 | 9.69 | 1.13 | 9.78 | 9.39 | -0.39 |  |  |
|  | 37➝30, 33 °C | Fucose | 64.3 | 64.20, 65.7 | -0.1, 1.4 | - | - | - | ProA labelling, HILIC | Hennicke et al. (2019) |
|  |  | Galactose | - | - | - | - | - | - |  |  |
|  |  | Sialic acid | 28.4 | 28.2, 21.8 | -0.2, 1.4 | - | - | - |  |  |
|  | 37➝33 °C | Fucose | 100 | 100 | 0 | 103 | 100 | -3 | 2-AA labelling, HPLC | Kim et al. (2018) |
|  |  | Galactose | 14.56 | 17 | 2.44 | 25 | 28 | 3 |  |  |
|  |  | Sialic acid | 7.14 | 3.13 | -4.01 | 1 | 1 | 0 |  |  |
|  | 37➝30, 34 °C | Fucose | 98.2 | 97.25, 97.90 | -0.93, -0.3 | - | - | - | 2-AB labelling, UPLC | Madabhushi et al. (2021) |
|  |  | Galactose | 20.9 | 21.1, 22.2 | 0.2, 1.3 | - | - | - |  |  |
|  |  | Sialic acid |  |  |  | - | - | - |  |  |
|  | 37➝32 °C | Fucose | 96.97 | 90.45 | -6.52 | 91.3 | 86.2 | -5.1 | SELDI TOF-MS | Tait et al. (2013) |
|  |  | Galactose | 24.16 | 23.14 | -1.02 | 38.95 | 37.6 | -1.35 |  |  |
|  |  | Sialic acid | 0 | 0 | 0 | 0 | 0 | 0 |  |  |
|  | 36.5➝33.5, 35 °C | Fucose | 93.27 | 95.51, 96.41 | 2.24, 3.14 | 81 | 85, 84 | 4, 3 | Not specified | Yang & Ierapetritou (2021) |
|  |  | Galactose | 54.47 | 30.17, 11.49 | -24.3, -42.98 | 63.8 | 43.9, 17.8 | -19.9, -46 |  |  |
|  |  | Sialic acid | 13.47 | 6.54, 5.62 | -6.93, -7.55 | 9 | 3, 1 | -6, -8 |  |  |
|  | 36.5➝32 °C | Fucose | 94.02 | 90.87 | -3.15 | 92.8 | 88.6 | -4.2 | Capillary electrophoresis (LabChip GXII Instrument) | Sou et al. (2015) |
|  |  | Galactose | 19.05 | 9.85 | -9.2 | 34.1 | 17.9 | -16.2 |  |  |
|  |  | Sialic acid | 0 | 0 | 0 | 0 | 0 | 0 |  |  |
|  | 36.5➝32 °C | Fucose | 95.05 | 92.06 | -2.99 | 93.67 | 88.67 | -5 | Capillary electrophoresis (LabChip GXII Instrument) | Sou et al. (2017) |
|  |  | Galactose | 18.95 | 8.84 | -10.11 | 34.13 | 16.86 | -17.27 |  |  |
|  |  | Sialic acid | 0 | 0 | 0 | 0 | 0 | 0 |  |  |
|  | 34➝30 °C | Fucose | 97.43 | 95.07 | -2.36 | 87.3 | 84.9 | -2.4 | 2-AB labelling, HILIC | Mellahi et al. (2019) |
|  |  | Galactose | 49.92 | 48.12 | -1.8 | 66.8 | 66.2 | -0.6 |  |  |
|  |  | Sialic acid | 8.87 | 8.98 | 0.11 | 5.7 | 5.2 | -0.5 |  |  |
|  | 37➝32 °C | Fucose | 96.12 | 95.37 | -0.75 | 86.7 | 88.5 | 1.8 | 2-AB labelling, UPLC | McHugh et al. (2020) |
|  |  | Galactose | 6.71 | 4.9 | -1.81 | 11.5 | 8.8 | -2.7 |  |  |
|  |  | Sialic acid | 0 | 0 | 0 | 0 | 0 | 0 |  |  |
| **Periodic shift** | | | | | | | | | | |
|  | 37⟷33 °C | Fucose | 100 | 100, 100 | 0, 0 | 100 | 99, 99 | -1, -1 | 2-AA labelling, HPLC | Kim et al. (2018) |
|  |  | Galactose | 14.56 | 18.69, 16.67 | 4.13, 2.11 | 25 | 31, 27 | 6, 2 |  |  |
|  |  | Sialic acid | 7.14 | 9.09, 6.90 | 1.92, -0.24 | 1 | 2, 2 | 1, 1 |  |  |

Table S3. Calculated impact of culture pH manipulations on glycan indices (GIx), glycan indices difference (∆GIx) and summative indices, and described method for glycan analysis of therapeutic IgG-related products in CHO cell systems.

| Kind of manipulation | | Sugar motive | GIx control, [%] | GIx manipulation, [%] | ∆GIx, [%] | Summative index control, [%] | Summative index manipulation, [%] | ∆Summative index, [%] | Method of glycan analysis | Reference |
| --- | --- | --- | --- | --- | --- | --- | --- | --- | --- | --- |
| ***pH*** | | | | | | | | | | |
| **Without pH control** | | | | | | | | | | |
|  | No control | Fucose | 82.1 | 85.4 | 3.3 | - | - | - | MALDI TOF-MS | Maralingannavar et al. (2018) |
|  |  | Galactose | 30.4 | 30.1 | -0.3 | - | - | - |  |  |
|  |  | Sialic acid | - | - | - | - | - | - |  |  |
|  | No control | Fucose | 100 | 100 | 0 | 99.6 | 100 | 0.4 | 2-AA labelling, HPLC | Kim et al. (2018) |
|  |  | Galactose | 23.42 | 26.13 | 2.71 | 33.4 | 36.8 | 3.4 |  |  |
|  |  | Sialic acid | 5.19 | 4.47 | -0.72 | 2.1 | 1.6 | -0.5 |  |  |
|  | No control, bioreactor | Fucose | 100 | 100 | 0 | 79 | 78.4 | -0.6 | HILIC, LC-MS/MS | Monteil et al. (2016) |
|  |  | Galactose | 22.78 | 22.83 | 0.05 | 30.9 | 27.2 | -3.7 |  |  |
|  |  | Sialic acid | 0 | 0 | 0 | 0 | 0 | 0 |  |  |
|  | No control, shake flask | Fucose | 100 | 100 | 0 | 78.8 | 80.5 | 1.7 | HILIC, LC-MS/MS | Monteil et al. (2016) |
|  |  | Galactose | 27.28 | 23.98 | -3.3 | 37.9 | 33.1 | -4.8 |  |  |
|  |  | Sialic acid | 0 | 0 | 0 | 0 | 0 | 0 |  |  |
| **Constant higher setpoint** | | | | | | | | | | |
|  | 7.2 | Fucose | 96.46 | 95.25 | -1.21 | - | - | - | 2-AB labelling, UPLC | Brunner et al. (2017) |
|  |  | Galactose | 27.86 | 29.9 | 2.04 | - | - | - |  |  |
|  |  | Sialic acid | 0.99 | 1.6 | 0.61 | - | - | - |  |  |
|  | 7.2, 7.4 | Fucose | 100 | 100 | 100 | 99.6 | 99.5, 100 | -0.1, 0.4 | 2-AA labelling, HPLC | Kim et al. (2018) |
|  |  | Galactose | 23.42 | 19.98, 13.86 | -3.44, -9.56 | 33.4 | 27.6, 19.2 | -5.8, -14.2 |  |  |
|  |  | Sialic acid | 5.19 | 4.74, 5.34 | -0.45, 0.15 | 2.1 | 1.70, 1.4 | -0.4, -0.7 |  |  |
|  | 7.2 | Fucose | 98.35 | 98.45 | 0.1 | - | - | - | 2-AB labelling, UPLC | Madabhushi et al. (2021) |
|  |  | Galactose | 20.9 | 26.5 | 5.6 | - | - | - |  |  |
|  |  | Sialic acid | - | - | - | - | - | - |  |  |
|  | 7.1 | Fucose | 97.75 | 97.42 | -0.33 | 97.81 | 98.65 | 0.84 | UPLC-HILIC-FLR | Lee et al. (2021) |
|  |  | Galactose | 8.5 | 9.13 | 0.63 | 15.86 | 17.01 | 1.15 |  |  |
|  |  | Sialic acid | 2.55 | 2.62 | 0.07 | 0.43 | 0.48 | 0.05 |  |  |
|  | 7.03-7.24 | Fucose | - | - | - | - | - | - | 2-AB labelling, UPLC | Jiang et al. (2018) |
|  |  | Galactose | 21.7 | 13.1-23.4 | -8.6-1.7 | - | - | - |  |  |
|  |  | Sialic acid | - | - | - | - | - | - |  |  |
| **Constant lower setpoint** | | | | | | | | | | |
|  | 6.8 | Fucose | 96.46 | 96.48 | 0.02 | - | - | - | 2-AB labelling, UPLC | Brunner et al. (2017) |
|  |  | Galactose | 27.86 | 25 | -2.86 | - | - | - |  |  |
|  |  | Sialic acid | 0.99 | 0.84 | -0.15 | - | - | - |  |  |
|  | 6.8 | Fucose | 100 | 100 | 0 | 66.3 | 72.7 | 6.4 | 2-AB labelling, HILIC | Aghamohseni et al. (2014) |
|  |  | Galactose | 71.37 | 77.03 | 5.66 | 49.3 | 57.9 | 8.6 |  |  |
|  |  | Sialic acid | 9.69 | 15.61 | 5.92 | 6.9 | 13.5 | 6.6 |  |  |
|  | 6.6, 6.8 | Fucose | 100 | 100 | 0 | 99.6 | 100, 100 | 0.4, 0.4 | 2-AA labelling, HPLC | Kim et al. (2018) |
|  |  | Galactose | 23.42 | 27.13, 23,72 | 3.71, -3.42 | 33.4 | 39.1, 34.7 | 5.7, 1.3 |  |  |
|  |  | Sialic acid | 5.19 | 2.16, 2.40 | -3.03, 0.25 | 2.1 | 1, 1 | -1.1, -1.1 |  |  |
|  | 6.8 | Fucose | 98.35 | 98.25 | -0.1 | - | - | - | 2-AB labelling, UPLC | Madabhushi et al. (2021) |
|  |  | Galactose | 20.9 | 20.8 | -0.1 | - | - | - |  |  |
|  |  | Sialic acid | - | - | - | - | - | - |  |  |
|  | 6.7 | Fucose | 97.75 | 96.37 | -1.38 | 97.81 | 95.47 | -2.34 | UPLC-HILIC-FLR | Lee et al. (2021) |
|  |  | Galactose | 8.5 | 6.18 | -2.32 | 15.86 | 11.44 | -4.42 |  |  |
|  |  | Sialic acid | 2.55 | 3.24 | 0.69 | 0.43 | 0.39 | -0.04 |  |  |
|  | 6.78-6.97 | Fucose | - | - | - | - | - | - | 2-AB labelling, UPLC | Jiang et al. (2018) |
|  |  | Galactose | 21.7 | 11.10-15.5 | -10.60--6.2 | - | - | - |  |  |
|  |  | Sialic acid | - | - | - | - | - | - |  |  |
| **Shift to different setpoint** | | | | | | | | | | |
|  | 7.8➝6.8 | Fucose | 100 | 100 | 0 | 66.3 | 77 | 10.7 | 2-AB labelling, HILIC | Aghamohseni et al. (2014) |
|  |  | Galactose | 71.37 | 71.01 | -0.36 | 49.3 | 58.2 | 8.9 |  |  |
|  |  | Sialic acid | 9.7 | 9.9 | 0.2 | 6.9 | 8.5 | 1.6 |  |  |
|  | 7.05➝6.75, 6.90 | Fucose | 64.3 | 62 | 65.8 | - | - | - | ProA labelling, HILIC | Hennicke et al. (2019) |
|  |  | Galactose | - | - | - | - | - | - |  |  |
|  |  | Sialic acid | 28.4 | 32.4 | 37.9 | - | - | - |  |  |
|  | 7.0➝6.8, 6.9 | Fucose | 93.07 | 93.32, 94.09 | 0.25, 1.02 | 79.7 | 80.2, 82.1 | 0.5, 2.4 | Not specified | Yang & Ierapetritou (2021) |
|  |  | Galactose | 48.76 | 49.28, 51 | 0.52, 2.24 | 63.2 | 63.9, 66.3 | 0.7, 3.1 |  |  |
|  |  | Sialic acid | 10.18 | 10.39, 11.12 | 0.21, 0.94 | 8.5 | 8.8, 9.9 | 0.3, 1.4 |  |  |
|  | 7.15➝6.7-7.0 | Fucose | 98.76 | 98.86, 98.97, 98.98 | 0.1, 0.21, 0.22 | 95.3 | 95.9, 96.4, 96.9 | 0.6, 1.1, 1.6 | Not specified | Villiger et al. (2016) |
|  |  | Galactose | 15.54 | 19.38, 21.46, 24.77 | 3.84, 8.92, 9.23 | 27.3 | 33.8, 36.8, 42.3 | 6.5, 3, 15 |  |  |
|  |  | Sialic acid | 0.67 | 0, 2.39, 2.27 | -0.67, 1.72, 1.62 | 0.2 | 0, 0.7, 0.8 | -0.2, 0.5, 0.6 |  |  |
|  | 6.95➝6.75 | Fucose | 89.99 | 90.39 | 0.4 | 87.2 | 87.5 | 0.3 | UPLC, LC-MS | Xie et al. (2016) |
|  |  | Galactose | 18.01 | 12.97 | -5.04 | 30.3 | 22.3 | -8 |  |  |
|  |  | Sialic acid | 0 | 0 | 0 | 0 | 0 | 0 |  |  |
|  | 7.15➝6.85 | Fucose | 92.76 | 86.34 | -6.42 | 93.5 | 85.3 | -8.2 | 2-AB labelling, HPLC | Zheng et al. (2018) |
|  |  | Galactose | 21.13 | 27.99 | 6.86 | 39.1 | 48.4 | 9.3 |  |  |
|  |  | Sialic acid | 0 | 0 | 0 | 0 | 0 | 0 |  |  |
| **Oscillatory pH** | | | | | | | | | | |
|  | 6.9⟷7.3 | Fucose | 85.3 | 84.9, 84.5, 85.5 | -0.4, -0.8, 0.2 | - | - | - | RP-HPLC, QTOF-MS | Zakrzewski et al. (2022) |
|  |  | Galactose | 12.6 | 8, 5.7, 6.5 | -4.6, -6.9, -6.1 | - | - | - |  |  |
|  |  | Sialic acid | - | - | - | - | - | - |  |  |

Table S4. Calculated impact of culture dissolved oxygen and CO_2_ partial pressure manipulations on glycan indices (GIx), glycan indices difference (∆GIx) and summative indices, and described method for glycan analysis of therapeutic IgG-related products in CHO cell systems.

| Kind of manipulation | | Sugar motive | GIx control, [%] | GIx manipulation, [%] | ∆GIx, [%] | Summative index control, [%] | Summative index manipulation, [%] | ∆Summative index, [%] | Method of glycan analysis | Reference |
| --- | --- | --- | --- | --- | --- | --- | --- | --- | --- | --- |
| ***Dissolved oxygen*** | | | | | | | | | | |
| **Constant higher setpoint** | | | | | | | | | | |
|  | 40% DOT | Fucose | 96.47 | 95.25 | -1.22 | - | - | - | 2-AB labelling, UPLC | Brunner et al. (2017) |
|  |  | Galactose | 27.72 | 27.8 | 0.08 | - | - | - |  |  |
|  |  | Sialic acid | 0.99 | 1.125 | 0.135 | - | - | - |  |  |
|  | 50% DOT | Fucose | 96 | 96 | 0 | - | - | - | RP-HPLC, QTOF-MS | Zakrzewski et al. (2022) |
|  |  | Galactose | 20 | 22.5 | 2.5 | - | - | - |  |  |
|  |  | Sialic acid | - | - | - | - | - | - |  |  |
| **Constant lower setpoint** | | | | | | | | | | |
|  | 10% DOT | Fucose | 96.47 | 96.5 | 0.03 | - | - | - | 2-AB labelling, UPLC | Brunner et al. (2017) |
|  |  | Galactose | 27.72 | 26.7 | -1.02 | - | - | - |  |  |
|  |  | Sialic acid | 0.99 | 1.15 | 0.16 | - | - | - |  |  |
|  | 10% DOT | Fucose | 98.35 | 98.35 | 0 | - | - | - | 2-AB labelling, UPLC | Madabhushi et al. (2021) |
|  |  | Galactose | 20.9 | 20.6 | -0.3 | - | - | - |  |  |
|  |  | Sialic acid | - | - | - | - | - | - |  |  |
|  | 10% DOT | Fucose | 90.91 | 90.66 | -0.25 | - | - | - | RP-HPLC, QTOF-MS | Zakrzewski et al. (2022) |
|  |  | Galactose | 10.49 | 13.49 | 3 | - | - | - |  |  |
|  |  | Sialic acid | - | - | - | - | - | - |  |  |
| **Oscillatory DOT** | | | | | | | | | | |
|  | 8⟷37% DOT | Fucose | 96 | 97, 97, 96.5 | 1, 1, 0.5 | - | - | - | RP-HPLC, QTOF-MS | Zakrzewski et al. (2022) |
|  |  | Galactose | 20 | 19.5, 18, 19.5 | -0.5, -2, -0.5 | - | - | - |  |  |
|  |  | Sialic acid | - | - | - | - | - | - |  |  |
| ***Other*** | | | | | | | | | | |
| **Antioxidant addition** | | | | | | | | | | |
|  | 100 μM baicalein | Fucose | 100 | 100 | 0 | 99.3 | 99.4 | 0.1 | LC-MS | Ha et al. (2018) |
|  |  | Galactose | 13.85 | 19.52 | 5.67 | 23.3 | 32.8 | 9.5 |  |  |
|  |  | Sialic acid | - | - | - | - | - | - |  |  |
|  | 15 mM S-sulfocysteine | Fucose | 100 | 100 | 0 | 93.6 | 90.8 | -2.8 | 2-AB labelling, HPLC | Hecklau et al. (2016) |
|  |  | Galactose | 15.67 | 17.96 | 2.29 | 27.6 | 30.8 | 3.2 |  |  |
|  |  | Sialic acid | - | - | - | - | - | - |  |  |
| **Higher *p*CO2 setpoint** | | | | | | | | | | |
|  | 12.5, 20% pCO2 | Fucose | 96.2 | 96.47, 96.8 | 0.27, 0.6 | **-** | **-** | - | 2-AB labelling, UPLC | Brunner et al. (2017) |
|  |  | Galactose | 29 | 27.72, 24.6 | -1.28, -4.4 | **-** | **-** | - |  |  |
|  |  | Sialic acid | 1.23 | 0.99, 0.96 | -0.24, -0.27 | - | - | - |  |  |

Table S5. Calculated impact of osmolality manipulations on glycan indices (GIx), glycan indices difference (∆GIx) and summative indices, and described method for glycan analysis of therapeutic IgG-related products in CHO cell systems.

| Kind of manipulation | | Sugar motive | GIx control, [%] | GIx manipulation, [%] | ∆GIx, [%] | Summative index control, [%] | Summative index manipulation, [%] | ∆Summative index, [%] | Method of glycan analysis | Reference |
| --- | --- | --- | --- | --- | --- | --- | --- | --- | --- | --- |
| ***Osmolality*** | | | | | | | | | | |
| **Higher osmolality** | | | | | | | | | | |
|  | +100 mOsm/kg | Fucose | 84.46 | 74.01 | -10.45 | 45.5 | 43.2 | -2.3 | 2-AA labelling, UPLC | Lee et al. (2017b) |
|  |  | Galactose | 85.14 | 78.4 | -6.74 | 44.8 | 42.5 | -2.3 |  |  |
|  |  | Sialic acid | - | - | - | - | - | - |  |  |
|  | +100, 200 mOsm/kg | Fucose | 98.35 | 97.75, 96.45 | -0.6, -1.9 | - | - | - | 2-AB labelling, UPLC | Madabhushi et al. (2021) |
|  |  | Galactose | 20.9 | 18.2, 17.3 | -2.7, -3.6 | - | - | - |  |  |
|  |  | Sialic acid | - | - | - | - | - | - |  |  |
|  | +60, 120, 180 mOsm/kg | Fucose | 96.13 | 98.4, 96.82, 97.55 | 2.27, 0.69, 1.42 | - | - | - | 2-AB labelling, HPLC | Qin et al. (2019) |
|  |  | Galactose | 14.74 | 3.37, 3.64, 3.48 | -11.37, -11.1, -11.26 | - | - | - |  |  |
|  |  | Sialic acid | - | - | - | - | - | - |  |  |
|  | +75, 170 mOsm/kg | Fucose | - | - | - | - | - | - | 2-AB labelling, UPLC | Jiang et al. (2018) |
|  |  | Galactose | 12.57 | 9.97, 10.84 | -2.6, -1.73 | - | - | - |  |  |
|  |  | Sialic acid | - | - | - | - | - | - |  |  |
|  | +370, 420, 470 mOsm/kg | Fucose | 95.53 | 94.92, 94.97, 94.94 | -0.61, -0.56, -0.59 | 96.2 | 95.3, 96.2, 93.8 | -3.9, 0, -2.4 | APTS labelling, capillary electrophoresis (SCIEX) | Alhuthali et al. (2021) |
|  |  | Galactose | 17.87 | 17.08, 13.82, 11.64 | -0.79, -4.05, -6.23 | 32.2 | 30.5, 25.4, 21.5 | -1.7, -6.8, -10.7 |  |  |
|  |  | Sialic acid | - | - | - | - | - | - |  |  |
|  | +410, 460, 500 mOsm/kg | Fucose | 95.53 | 95.55, 92.57, 85.26 | 0.02, -2.96, -10.27 | 96.2 | 96.7, 93.4, 86.2 | 0.5, -2.8, -10 | APTS labelling, capillary electrophoresis (SCIEX) | Alhuthali et al. (2021) |
|  |  | Galactose | 17.87 | 8.7, 10.36, 12.91 | -9.17, -7.51, -4.96 | 32.2 | 16.5, 19.4, 23.8 | -15.7, -12.8, -8.4 |  |  |
|  |  | Sialic acid | - | - | - | - | - | - |  |  |
|  | +60, 120, 180 mOsm/kg | Fucose | 90.91 | 91.38, 92.69, 90.17 | 0.47, 1.78, -0.74 | - | - | - | 2-AB labelling, HPLC | Qin et al. (2019) |
|  |  | Galactose | 54.95 | 36.08, 38.71, 40 | -18.87, -16.24, -14.95 | - | - | - |  |  |
|  |  | Sialic acid | - | - | - | - | - | - |  |  |

Table S6. Calculated impact of chemical additives supplementation on glycan indices (GIx), glycan indices difference (∆GIx) and summative indices, and described method for glycan analysis of therapeutic IgG-related products in CHO cell systems.

| Kind of manipulation | | Sugar motive | GIx control, [%] | GIx manipulation, [%] | ∆GIx, [%] | Summative index control, [%] | Summative index manipulation, [%] | ∆Summative index, [%] | Method of glycan analysis | Reference |
| --- | --- | --- | --- | --- | --- | --- | --- | --- | --- | --- |
| ***Chemical additives*** | | | | | | | | | | |
| **HDAC inhibitor addition** | | | | | | | | | | |
|  | 0.2, 1, 2, 4 mM NaBu | Fucose | 100 | 100, 100, 100, 100 | 0, 0, 0, 0 | 96.3 | 94.7, 93.8, 94.4, 95.7 | -1.6, -2.5, -1.9, -0.6 | APTS labelling, capillary electrophoresis | Hong et al. (2014) |
|  |  | Galactose | 17.03 | 13.20, 10.86, 10.92, 10.45 | -4.10, -6.17, -6.11, -6.58 | 29.2 | 22.3, 17.6, 18.4, 18.8 | -6.9, -11.6, -10.8, -10.4 |  |  |
|  |  | Sialic acid | - | - | - | - | - | - |  |  |
|  | 5 mM NaBu | Fucose | 98.24 | 98.43 | 0.19 | 94.8 | 94.3 | -0.5 | 2-AB labelling, UPLC | Madabhushi et al. (2021) |
|  |  | Galactose | 10.98 | 10.13 | -0.85 | 20.1 | 18.4 | -1.7 |  |  |
|  |  | Sialic acid | - | - | - | - | - | - |  |  |
|  | 3.5 mM valproic acid | Fucose | 100 | 100 | 0 | 80.8 | 86.9 | 6.1 | 2-AB labelling, HILIC-UPLC | Yang et al. (2014) |
|  |  | Galactose | 10.52 | 12.08 | 1.56 | 15 | 18.8 | 3.8 |  |  |
|  |  | Sialic acid | - | - | - | - | - | - |  |  |
|  | 1.5 mM valeric acid | Fucose | 100 | 100 | 0 | 16.8 | 15.1 | -1.7 | UPLC, Q TOF-MS | Park et al. (2016) |
|  |  | Galactose | 21.43 | 28.15 | 6.72 | 6.8 | 8.2 | 1.4 |  |  |
|  |  | Sialic acid | - | - | - | - | - | - |  |  |
| **qP-enhancing chemical addition** | | | | | | | | | | |
|  | 5 μM CDK4/6 inhibitor | Fucose | 91.03 | 96.43 | 5.4 | 100 | 86.4 | -13.6 | 2-AA labelling, HILIC | Du et al. (2015) |
|  |  | Galactose | 22 | 24.61 | 2.61 | 45.3 | 39.3 | -6 |  |  |
|  |  | Sialic acid | - | - | - | - | - | - |  |  |
|  | 50 μM BIX and 1%v/v DMSO | Fucose | 100 | 100 | 0 | 99.5 | 99.8 | 0.3 | 2-AB labelling, HPLC | Ha et al. (2019) |
|  |  | Galactose | 19.95 | 23.75 | 3.8 | 35.9 | 42.3 | 6.4 |  |  |
|  |  | Sialic acid | - | - | - | - | - | - |  |  |
|  | 5 μM foskolin | Fucose | 97.19 | 96.9 | -0.29 | 96.7 | 96.8 | 0.1 | UPLC | Yoon et al. (2020) |
|  |  | Galactose | 24.02 | 25.13 | 1.11 | 41.8 | 43.6 | 1.8 |  |  |
|  |  | Sialic acid | - | - | - | - | - | - |  |  |

**SUPPLEMENTARY REFERENCES**

Aghamohseni, H., Ohadi, K., Spearman, M., Krahn, N., Moo-Young, M., Scharer, J. M., Butler, M., & Budman, H. M. (2014). Effects of nutrient levels and average culture pH on the glycosylation pattern of camelid-humanized monoclonal antibody. *Journal of Biotechnology*, *186*, 98–109. https://doi.org/10.1016/J.JBIOTEC.2014.05.024

Aghamohseni, H., Spearman, M., Ohadi, K., Braasch, K., Moo-Young, M., Butler, M., & Budman, H. M. (2017). A semi-empirical glycosylation model of a camelid monoclonal antibody under hypothermia cell culture conditions. *Journal of Industrial Microbiology and Biotechnology*, *44*(7), 1005–1020. https://doi.org/10.1007/S10295-017-1926-Z

Alhuthali, S., Kotidis, P., & Kontoravdi, C. (2021). Osmolality effects on cho cell growth, cell volume, antibody productivity and glycosylation. *International Journal of Molecular Sciences*, *22*(7). https://doi.org/10.3390/ijms22073290

Blondeel, E. J. M., & Aucoin, M. G. (2018). Supplementing glycosylation: A review of applying nucleotide-sugar precursors to growth medium to affect therapeutic recombinant protein glycoform distributions. *Biotechnology Advances*, *36*(5), 1505–1523. https://doi.org/10.1016/J.BIOTECHADV.2018.06.008

Brunner, M., Fricke, J., Kroll, P., & Herwig, C. (2017). Investigation of the interactions of critical scale-up parameters (pH, pO2 and pCO2) on CHO batch performance and critical quality attributes. *Bioprocess and Biosystems Engineering*, *40*(2), 251–263. https://doi.org/10.1007/S00449-016-1693-7/TABLES/5

Du, Z., Treiber, D., Mccarter, J. D., Fomina-Yadlin, D., Saleem, R. A., Mccoy, R. E., Zhang, Y., Tharmalingam, T., Leith, M., Follstad, B. D., Dell, B., Grisim, B., Zupke, C., Heath, C., Morris, A. E., & Reddy, P. (2015). Use of a small molecule cell cycle inhibitor to control cell growth and improve specific productivity and product quality of recombinant proteins in CHO cell cultures. *Biotechnology and Bioengineering*, *112*(1), 141–155. https://doi.org/10.1002/BIT.25332

Galbraith, D. J., Tait, A. S., Racher, A. J., Birch, J. R., & James, D. C. (2006). Control of Culture Environment for Improved Polyethylenimine-Mediated Transient Production of Recombinant Monoclonal Antibodies by CHO Cells. *Biotechnology Progress*, *22*(3), 753–762. https://doi.org/10.1021/bp050339v

Ha, T. K., Hansen, A. H., Kildegaard, H. F., & Lee, G. M. (2019). BiP Inducer X: An ER Stress Inhibitor for Enhancing Recombinant Antibody Production in CHO Cell Culture. *Biotechnology Journal*, *14*(10), 1900130. https://doi.org/10.1002/BIOT.201900130

Ha, T. K., Hansen, A. H., Kol, S., Kildegaard, H. F., & Lee, G. M. (2018). Baicalein Reduces Oxidative Stress in CHO Cell Cultures and Improves Recombinant Antibody Productivity. *Biotechnology Journal*, *13*(3), 1700425. https://doi.org/10.1002/BIOT.201700425

Hecklau, C., Pering, S., Seibel, R., Schnellbaecher, A., Wehsling, M., Eichhorn, T., Hagen, J. von, & Zimmer, A. (2016). S-Sulfocysteine simplifies fed-batch processes and increases the CHO specific productivity via anti-oxidant activity. *Journal of Biotechnology*, *218*, 53–63. https://doi.org/10.1016/J.JBIOTEC.2015.11.022

Hennicke, J., Reinhart, D., Altmann, F., & Kunert, R. (2019). Impact of temperature and pH on recombinant human IgM quality attributes and productivity. *New Biotechnology*, *50*, 20–26. https://doi.org/10.1016/J.NBT.2019.01.001

Hong, J. K., Lee, S. M., Kim, K. Y., & Lee, G. M. (2014). Effect of sodium butyrate on the assembly, charge variants, and galactosylation of antibody produced in recombinant Chinese hamster ovary cells. *Applied Microbiology and Biotechnology*, *98*(12), 5417–5425. https://doi.org/10.1007/S00253-014-5596-8/METRICS

Jiang, R., Chen, H., & Xu, S. (2018). pH excursions impact CHO cell culture performance and antibody N-linked glycosylation. *Bioprocess and Biosystems Engineering*, *41*(12), 1731–1741. https://doi.org/10.1007/s00449-018-1996-y

Kim, S. M., Chang, K. H., & Oh, D. J. (2018). Effect of Environmental Parameters on Glycosylation of Recombinant Immunoglobulin G Produced from Recombinant CHO Cells. *Biotechnology and Bioprocess Engineering 2018 23:4*, *23*(4), 456–464. https://doi.org/10.1007/S12257-018-0109-8

Lee, A. P., Kok, Y. J., Lakshmanan, M., Leong, D., Zheng, L., Lim, H. L., Chen, S., Mak, S. Y., Ang, K. S., Templeton, N., Salim, T., Wei, X., Gifford, E., Tan, A. H. M., Bi, X., Ng, S. K., Lee, D. Y., Ling, W. L. W., & Ho, Y. S. (2021). Multi-omics profiling of a CHO cell culture system unravels the effect of culture pH on cell growth, antibody titer, and product quality. *Biotechnology and Bioengineering*, *118*(11), 4305–4316. https://doi.org/10.1002/BIT.27899

Lee, J. H., Jeong, Y. R., Kim, Y. G., & Lee, G. M. (2017b). Understanding of decreased sialylation of Fc-fusion protein in hyperosmotic recombinant Chinese hamster ovary cell culture: N-glycosylation gene expression and N-linked glycan antennary profile. *Biotechnology and Bioengineering*, *114*(8), 1721–1732. https://doi.org/10.1002/BIT.26284

Madabhushi, S. R., Podtelezhnikov, A. A., Murgolo, N., Xu, S., & Lin, H. (2021). Understanding the effect of increased cell specific productivity on galactosylation of monoclonal antibodies produced using Chinese hamster ovary cells. *Journal of Biotechnology*, *329*, 92–103. https://doi.org/10.1016/J.JBIOTEC.2021.01.023

Maralingannavar, V., Shenoy, B. R., Hazarika, J., Unnikrishnan, D., Prabhu, A., Maity, S., & Gadgil, M. (2018). In situ base release for pH maintenance can allow shake flasks to better mimic bioreactor performance for CHO cell culture. *Journal of Chemical Technology & Biotechnology*, *93*(10), 2842–2850. https://doi.org/10.1002/JCTB.5635

McHugh, K. P., Xu, J., Aron, K. L., Borys, M. C., & Li, Z. J. (2020). Effective temperature shift strategy development and scale confirmation for simultaneous optimization of protein productivity and quality in Chinese hamster ovary cells. *Biotechnology Progress*, *36*(3), e2959. https://doi.org/10.1002/BTPR.2959

Mellahi, K., Brochu, D., Gilbert, M., Perrier, M., Ansorge, S., Durocher, Y., & Henry, O. (2019). Assessment of fed-batch cultivation strategies for an inducible CHO cell line. *Journal of Biotechnology*, *298*, 45–56. https://doi.org/10.1016/J.JBIOTEC.2019.04.005

Monteil, D. T., Juvet, V., Paz, J., Moniatte, M., Baldi, L., Hacker, D. L., & Wurm, F. M. (2016). A comparison of orbitally-shaken and stirred-tank bioreactors: pH modulation and bioreactor type affect CHO cell growth and protein glycosylation. *Biotechnology Progress*, *32*(5), 1174–1180. https://doi.org/10.1002/BTPR.2328

Park, J. H., Noh, S. M., Woo, J. R., Kim, J. W., & Lee, G. M. (2016). Valeric acid induces cell cycle arrest at G1 phase in CHO cell cultures and improves recombinant antibody productivity. *Biotechnology Journal*, *11*(4), 487–496. https://doi.org/10.1002/BIOT.201500327

Qin, J., Wu, X., Xia, Z., Huang, Z., Zhang, Y., Wang, Y., Fu, Q., & Zheng, C. (2019). The effect of hyperosmolality application time on production, quality, and biopotency of monoclonal antibodies produced in CHO cell fed-batch and perfusion cultures. *Applied Microbiology and Biotechnology*, *103*(3), 1217–1229. https://doi.org/10.1007/S00253-018-9555-7/FIGURES/9

Sou, S. N., Jedrzejewski, P. M., Lee, K., Sellick, C., Polizzi, K. M., & Kontoravdi, C. (2017). Model-Based Investigation of Intracellular Processes Determining Antibody Fc-Glycosylation Under Mild Hypothermia. *Biotechnol. Bioeng*, *114*, 1570–1582. https://doi.org/10.1002/bit.26225/abstract

Sou, S. N., Sellick, C., Lee, K., Mason, A., Kyriakopoulos, S., Polizzi, K. M., & Kontoravdi, C. (2015). How Does Mild Hypothermia Affect Monoclonal Antibody Glycosylation? *Biotechnol. Bioeng*, *112*, 1165–1176. https://doi.org/10.1002/bit.25524/abstract

St. Amand, M. M., Hayes, J., Radhakrishnan, D., Fernandez, J., Meyer, B., Robinson, A. S., & Ogunnaike, B. A. (2016). Identifying a robust design space for glycosylation during monoclonal antibody production. *Biotechnology Progress*, *32*(5), 1149–1162. https://doi.org/10.1002/btpr.2316

Tait, A. S., Tarrant, R. D. R., Velez-Suberbie, M. L., Spencer, D. I. R., & Bracewell, D. G. (2013). Differential Response in Downstream Processing of CHO Cells Grown Under Mild Hypothermic Conditions. *Biotechnology Progress*, *29*(3), 688. https://doi.org/10.1002/BTPR.1726

Villiger, T. K., Scibona, E., Stettler, M., Broly, H., Morbidelli, M., & Soos, M. (2016). Controlling the time evolution of mAb N-linked glycosylation - Part II: Model-based predictions. *Biotechnology Progress*, *32*(5), 1135–1148. https://doi.org/10.1002/btpr.2315

Xie, P., Niu, H., Chen, X., Zhang, X., Miao, S., Deng, X., Liu, X., Tan, W. S., Zhou, Y., & Fan, L. (2016). Elucidating the effects of pH shift on IgG1 monoclonal antibody acidic charge variant levels in Chinese hamster ovary cell cultures. *Applied Microbiology and Biotechnology*, *100*(24), 10343–10353. https://doi.org/10.1007/S00253-016-7749-4/TABLES/3

Yang, O., & Ierapetritou, M. (2021). mAb Production Modeling and Design Space Evaluation Including Glycosylation Process. *Processes 2021, Vol. 9, Page 324*, *9*(2), 324. https://doi.org/10.3390/PR9020324

Yang, W. C., Lu, J., Nguyen, N. B., Zhang, A., Healy, N. V., Kshirsagar, R., Ryll, T., & Huang, Y. M. (2014). Addition of valproic acid to CHO cell fed-batch cultures improves monoclonal antibody titers. *Molecular Biotechnology*, *56*(5), 421–428. https://doi.org/10.1007/S12033-013-9725-X

Yoon, C., Kim, D., Lim, J. H., & Lee, G. M. (2020). Forskolin Increases cAMP Levels and Enhances Recombinant Antibody Production in CHO Cell Cultures. *Biotechnology Journal*, *15*(10), 2000264. https://doi.org/10.1002/BIOT.202000264

Zakrzewski, R., Lee, K., & Lye, G. J. (2022). Development of a miniature bioreactor model to study the impact of pH and DOT fluctuations on CHO cell culture performance as a tool to understanding heterogeneity effects at large-scale. *Biotechnology Progress*, *38*(4). https://doi.org/10.1002/btpr.3264

Zheng, C., Zhuang, C., Chen, Y., Fu, Q., Qian, H., Wang, Y., Qin, J., Wu, X., & Qi, N. (2018). Improved process robustness, product quality and biological efficacy of an anti-CD52 monoclonal antibody upon pH shift in Chinese hamster ovary cell perfusion culture. *Process Biochemistry*, *65*, 123–129. https://doi.org/10.1016/j.procbio.2017.11.013
